# Sequenced-based GWAS for linear classification traits in Belgian Blue beef cattle reveals new coding variants in genes regulating body size in mammals

**DOI:** 10.1186/s12711-023-00857-4

**Published:** 2023-11-28

**Authors:** José Luis Gualdrón Duarte, Can Yuan, Ann-Stephan Gori, Gabriel C. M. Moreira, Haruko Takeda, Wouter Coppieters, Carole Charlier, Michel Georges, Tom Druet

**Affiliations:** 1 Unit of Animal Genomics, GIGA-R & Faculty of Veterinary Medicine, University of Liège, Avenue de l’Hôpital, 1, Liège, 4000 Belgium; 2 Walloon Breeders Association, Rue des Champs Elysées, 4, 5590 Ciney, Belgium; 3 GIGA Genomic Platform, GIGA-R, University of Liège, Avenue de l’Hôpital, 1, 4000 Liège, Belgium

## Abstract

**Background:**

Cohorts of individuals that have been genotyped and phenotyped for genomic selection programs offer the opportunity to better understand genetic variation associated with complex traits. Here, we performed an association study for traits related to body size and muscular development in intensively selected beef cattle. We leveraged multiple trait information to refine and interpret the significant associations.

**Results:**

After a multiple-step genotype imputation to the sequence-level for 14,762 Belgian Blue beef (BBB) cows, we performed a genome-wide association study (GWAS) for 11 traits related to muscular development and body size. The 37 identified genome-wide significant quantitative trait loci (QTL) could be condensed in 11 unique QTL regions based on their position. Evidence for pleiotropic effects was found in most of these regions (e.g., correlated association signals, overlap between credible sets (CS) of candidate variants). Thus, we applied a multiple-trait approach to combine information from different traits to refine the CS. In several QTL regions, we identified strong candidate genes known to be related to growth and height in other species such as *LCORL-NCAPG* or *CCND2*. For some of these genes, relevant candidate variants were identified in the CS, including three new missense variants in *EZH2*, *PAPPA2* and *ADAM12*, possibly two additional coding variants in *LCORL*, and candidate regulatory variants linked to *CCND2* and *ARMC12*. Strikingly, four other QTL regions associated with dimension or muscular development traits were related to five (recessive) deleterious coding variants previously identified.

**Conclusions:**

Our study further supports that a set of common genes controls body size across mammalian species. In particular, we added new genes to the list of those associated with height in both humans and cattle. We also identified new strong candidate causal variants in some of these genes, strengthening the evidence of their causality. Several breed-specific recessive deleterious variants were identified in our QTL regions, probably as a result of the extreme selection for muscular development in BBB cattle.

**Supplementary Information:**

The online version contains supplementary material available at 10.1186/s12711-023-00857-4.

## Background

Reference populations that are built to implement genomic selection [1] in livestock species are valuable resources to understand the genetic basis for variation in complex traits. The size of these cohorts of genotyped and phenotyped individuals is increasing over the years, while genomic selection is applied to more and more livestock species and breeds, e.g. [2, 3]. Although data collection focuses mainly on traits of agronomic importance, these phenotypes might also help to study complex traits of interest for other applications. For instance, these populations can be used to study traits related to health such as fertility (in particular, in the context of artificial reproductive technologies that are massively used in livestock), or to fundamental biological processes such as meiotic recombination, e.g. [4, 5]. Traits recorded in multiple species allow to understand to what extent the genetic architecture of complex traits is conserved across species or how it evolves, e.g. [6]. A typical example would be the study of stature in mammals, where results obtained in humans, dog, cattle and horse have already been compared, revealing that genome-wide association signals are enriched in genes associated in other species, e.g. [6–8]. Indeed, association studies and scans for signatures of selection identified genes associated with height in multiple species such as *IGF1*, *PLAG1* or *LCORL-NACPG* [7, 9–13]. Similarly, variations in the *myostatin* (*MSTN*) gene that affect muscular development have been identified in several species including cattle, sheep, dog, horse, pig and humans [11, 12, 14–16]. Interestingly, livestock provides information on these complex traits in populations under intensive selection and with reduced effective population size.

The Belgian Blue beef (BBB) cattle breed represents an example of a breed intensively selected for muscular development. As a result, a loss-of-function mutation in the *MSTN* gene causing the double muscling phenotype [14] has been fixed through selection [14, 17]. However, additional genetic variation for muscular development is still available within the breed and has been exploited to further increase this trait [17]. In addition, several recessive deleterious variants under balancing selection were segregating in the population at high frequency before the implementation of genetic tests, including a 2-bp deletion in the open reading frame of the *MRC2* gene [18] and a splice-site variant in the *RNF11* gene [19]. For these loss-of-function (LoF) variants, heterozygote advantage resulted from the favorable effect of these alleles on selected traits such as muscular development or stature. Similarly, an R844Q missense variant in the *WWP1* gene presented evidence for both a recessive deleterious effect and a favorable effect on muscular development [20]. Indeed, significantly fewer homozygotes than expected were observed in the population in spite of a relatively high frequency, indicating selection against homozygotes and a recessive effect. In the last years, a genomic selection program has been implemented in BBB cattle, with the genotyping of individuals having started in 2016 [21]. The reference population is phenotyped mainly for a set of linear classification traits, related to body size and muscular development, that are routinely recorded on adult females [21]. This population represents an example of a cattle breed that is intensively selected for muscular development, and for body size to a lesser extent.

Genome-wide association studies (GWAS) are one of the main tools to decipher the architecture of complex traits. Genomic selection reference populations (consisting in individuals with both genotypes and phenotypes) offer the opportunity to apply such scans in livestock species, but the marker density is often too low to capture well all causal variants and to perform the fine-mapping. Therefore, genotype imputation to the sequence level thanks to a reference panel of sequenced individuals [22, 23] is a recommended practice. Such sequence-based GWAS have already been successfully implemented in cattle [8, 24–26]. In addition to sequence-based level approaches, multiple traits association methods [27, 28] might be useful to improve the fine-mapping resolution since subsets of recorded traits are often genetically correlated and affected by shared pleiotropic variants.

In this study, we performed such a sequence-based GWAS in BBB cattle for linear classification traits related to body size and muscular development. Multiple-traits information was leveraged to refine the set of candidate variants and to interpret the relation between associations for different quantitative trait loci (QTL) mapping to the same genomic region. For several of the identified QTL, we found genes that are associated to stature in other species among the candidate genes, providing further support for the presence of common genes regulating body size in mammals. For several of them, we identified coding variants as strong candidates that give stronger evidence of the causality of these genes. Besides these QTL, others were associated to five (recessive) deleterious variants that have favorable effects in the heterozygous state on traits related to muscular development.

## Methods

The analytical framework applied in our study, including the main steps described below, is summarized in Additional file 1: Fig. S1.

### Data

The “mapping population” of our study consisted in a set of 14,762 cows having both genotypes and phenotypes. These cows were genotyped on six different versions of Illumina BovineLD Genotyping BeadChips used by the EuroGenomics consortium [from 9983 to 20,502 single nucleotide polymorphisms (SNPs)], on EuroG MD Genotyping arrays (three versions with 51,809 to 57,979 SNPs), or on the Illumina BovineSNP50 DNA Analysis BeadChips (two versions, with 54,001 and 54,609 SNPs). Linear classification scores (assessed visually by a technician) for 10 traits including length, pelvis length, height, chest width, pelvis width, rib shape, rump, top muscling, shoulder muscling and buttock muscling (side and rear view) were available for 14,476 cows. In addition, height was measured at withers for 12,904 cows. These phenotypes were first pre-corrected for age effects with a quadratic regression and then corrected for the fixed effects from the genetic evaluation including a contemporary group effect (defined as a herd by date effect) and a correction for body condition score. Details on the phenotypes and the genetic evaluation are available on the website from the herd-book [29] and from the Walloon breeders association [30], and descriptive statistics are provided in Additional file 2: Table S1. In addition to the mapping population, other individuals were available as reference panel for genotype imputation. First, a set of 717 artificial insemination (AI) bulls was genotyped on the BovineHD DNA Analysis Kit. Among these, 658 were also genotyped on the Illumina BovineLD genotyping array. In addition, 199 animals, including 66 AI bulls, were genotyped on both Illumina BovineLD and BovineSNP50 genotyping arrays. Next, 9502 individuals without phenotype were genotyped on EuroG MD genotyping arrays. The number of individuals genotyped on the different arrays is described in Additional file 2: Table S2. Finally, whole-genome sequence data were available for 230 bulls at an average coverage depth of 35×, ranging from 11× to 68×.

### Read mapping and variant calling procedure

The sequencing data came from two distinct experiments. For a first group of 50 bulls previously described in Charlier et al. [20], DNA was extracted from sperm using standard procedures. PCR-free libraries were sequenced at the CNAG in Barcelona on an Illumina HiSeq 2000 with a paired-end protocol (2 × 100 bp), each sample being sequenced on multiple lanes. For the 180 remaining bulls, DNA was extracted from blood or sperm and paired-end sequencing (2 × 150 bp) was performed on an Illumina NovaSeq 6000 sequencer. Reads were aligned to the ARS-UCD1.2 (BosTau9) bovine genome assembly [31] with the BWA-MEM v0.7.5a software [32], sorted with Sambamba v0.6.6 [33] and PCR duplicates were marked with Picard Tools v2.7.1 [34]. BAM files were recalibrated using the GATK4 v4.1.7.0 software [31], using a list of 110,270,194 known variants provided as a resource by the 1000 Bull Genomes project [35, 36], and including variants from the run 7 of the project, as known polymorphic sites. Recalibrated BAM files from samples sequenced on different lanes at the CNAG were then merged per bull. Individual variant calling was performed with HaplotypeCaller (GATK4 v4.1.7.0) and the joint genotyping of all the genomic variant call format (GVCF) files was subsequently done with GenotypeGVCFs (GATK4 v4.1.7.0) in 5-Mb windows. Quality scores from the resulting VCF were then recalibrated using the variant quality score recalibration (VQSR) procedure with the VariantRecalibrator command (GATK4 v4.1.7.0) as recommended by the Broad Institute [37]. A set of 1,213,314 SNPs from all bovine commercial genotyping arrays available on the SNPchiMp v.3 server [38] was used as truth set, and the ~ 110 M SNPs provided by the 1000 Bull Genome (see above) project as known set. This procedure defines quality thresholds that would result in the conservation of different fractions of the truth set (e.g., 90, 95, 97.5%). Variants with a quality score below the 97.5 threshold, with a minor allele frequency (MAF) < 0.01, and multi-allelic sites were filtered out, resulting in a set of 12,830,339 SNPs and 2,502,613 indels.

### Marker selection and genotype imputation

Genotype imputation from low marker density (LMD) to the sequence level was performed in successive steps [39], using medium marker density (MMD) and high marker density (HMD) levels as intermediate steps. The LMD level consisted in all cows from the mapping populations genotyped on Illumina BovineLD arrays (11,521 cows). The reference population at the MMD level consisted in (i) cows from the mapping population and other individuals genotyped on EuroG MD arrays (12,475 animals) or genotyped on both Illumina BovineLD and Illumina Bovine50SNP arrays (467 individuals) and (ii) AI bulls genotyped simultaneously on Illumina BovineLD and Illumina BovineHD arrays (658 bulls). At the HMD level, the sequenced bulls or those genotyped on the Illumina BovineHD arrays defined the reference population, corresponding to 890 unique individuals. At each level, we selected markers that were common to all involved panels (for individuals genotyped on two arrays, selected markers had to be present on at least one of them) and that were useful for the imputation procedure (shared either with the previous or with the next level). We filtered out markers with a call rate lower than 0.95, with a MAF lower than 0.01, deviating from Hardy–Weinberg proportions (p < 0.001) or with more than 10 Mendelian inconsistencies (e.g., opposite homozygous genotypes in parent/offspring pairs), and individuals with a genotyping rate lower than 90%. As a result, we selected respectively 7525, 32,318 and 611,322 SNPs at the LMD, MMD and HMD levels. Beagle 4.1 [40] was first applied to the whole-genome sequence (WGS) and HMD reference panels to improve genotype calls and impute sporadically missing genotypes. Target and reference panels were phased with ShapeIT4.2 [41] and Minimac4 [42] was applied to achieve the imputation in the target panel. After each intermediary imputation step, we discarded SNPs with a MAF < 0.02 in the imputed set, and those with a reported imputation accuracy lower than 0.80 (the imputation accuracy obtained from Minimac4). In addition, we removed SNPs not useful for the next imputation step (for instance, SNP shared between LMD and MMD arrays but absent from the HMD array; these are useful for the first imputation step but no longer in the second step). As a result, we conserved 28,893 and 572,667 SNPs at the MMD and HMD level for imputation to the next level. These additional cleaning steps were applied to keep only SNPs that were expected to be accurately imputed. After the last imputation step, the final VCF file contained 11,537,240 SNPs and indels with a MAF > 0.01.

### Genome-wide association study

Single-trait GWAS (ST-GWAS) were performed on each trait using the following linear mixed model (LMM) approach with GEMMA [43] to test the association with marker $i$:

$$\mathbf{y}={\mathbf{1}}\mu +\mathbf{Z}\mathbf{g}+{\mathbf{x}}_{i}{{\upbeta }}_{i}+\mathbf{e},$$

where $\mathbf{y}$ is the vector of trait deviations, *µ* is the overall mean effect, $\mathbf{g}$ is a vector containing the random additive polygenic effects of the corresponding cows, ${{\upbeta }}_{i}$ is the additive effect of the tested SNP, ${\mathbf{x}}_{i}$ is a vector containing the allele dosage for the corresponding cows at marker $i$, $\mathbf{e}$ is a vector of random error terms and $\mathbf{Z}$ is an incidence matrix indicating which animal is associated with the phenotype. The covariance structure among the random polygenic effects $\mathbf{g}$ is a function of the genomic relationship matrix $\mathbf{G}$ obtained from the 28,893 SNPs from the MMD level and constructed using the first method proposed by VanRaden [44]. The number of independent tests per genome-scan was estimated with the procedure described in Druet et al. [17]. Briefly, we performed a genome-scan for association with height using a simple regression, providing us a distribution of uncorrected p-values. Then, we repeated genome-scans on 100,000 random permutations of the phenotypes and recorded the best p-value for each scan, providing the distribution of the best p-values under H0 that allowed us to obtain corrected p-values for the first scan. Finally, the number of independent tests was estimated to be equal to 500,900 (rounded to 500,000) based on the comparison of the uncorrected and corrected p-values and using the Sidak formula. As we repeated the association study for 11 traits, we also estimated the number of independent traits using the meff function (method = Galwey) from the poolR R package [45] and obtained a value of 7. As a result, we set the significance threshold to 1.43e−8 (− log_10_P > 7.84) after applying a Bonferroni correction for 3,500,000 independent tests. In regions where a significant QTL was detected, we also considered that other traits with significance levels below 1e−7 (− log_10_P > 7) presented evidence for association with a QTL in the region.

The set of candidate causal variants, referred to as credible sets (CS), were defined with two approaches. First, an iterative Bayesian step-wise selection (IBSS) approach implemented in SuSiE [46, 47], relying on summary statistics obtained from the ST-GWAS and on the linkage disequilibrium (LD) pattern among SNPs, was applied to identify a CS of SNPs with a probability higher than 0.95 of containing the causal variable (based on the individual posterior inclusion probabilities (PIP) from each SNP). This probability does not take into account the possibility that a causal variant is not included in the study (e.g., structural variants contributing to complex traits or variants excluded during the filtering process), or that some genotypes were incorrectly imputed. Note that the IBSS approach also provides multiple CS when several independent effects are detected in the QTL region. In addition, LD-based CS were obtained by selecting all the SNPs in high LD with the lead variants (with the minimal LD level r² set to 0.90 or 0.80).

### Comparison of associations across traits and multiple-trait association studies

For each trait, we identified genome-wide significant associations and considered other significant associations within 1-Mb as part of the same QTL region (QTLR). When significant SNPs were identified for different traits less than 1 Mb apart, the QTLR were considered as a single QTLR. To investigate whether associations for different traits in the same QTLR resulted from a pleiotropic QTL or from closely linked QTL, we compared the SNP significance levels obtained for pairs of traits. To that end, we selected all the SNPs located within 1 Mb around the lead SNP and computed correlations among t-values or signed p-values (on a − log10 scale) to take into account the effect direction [48, 49]. Furthermore, we excluded SNPs that were non-significant (p > 0.05) for both traits, as effects are not expected to be shared for these unassociated SNPs [49]. In addition, we studied overlap between CS obtained for all the traits presenting significant association in the QTLR to find further evidence of pleiotropy.

As we found evidence that several associations were shared across multiple traits, we decided to run multiple-trait GWAS (MT-GWAS) with the multivariate LMM approach implemented in GEMMA [50]. As recommended, we limited the number of phenotypes in the multivariate analyses. Therefore, we ran the model on two groups of six traits that were selected based on shared associations. The first group contained traits related mainly to body size (height, length, pelvis length, pelvis width, chest width, rib shape), whereas the second was more related to muscular development (shoulder and top muscling, side and rear-view of buttock muscling, rump and chest width). This MT-GWAS was used to combine information from multiple traits to improve the fine-mapping by defining multiple-trait LD-based CS. The MT-GWAS information was considered for fine-mapping when at least one of the six traits presented evidence of association (− log_10_P > 7). These correspond to association levels that would be genome-wide significant in a single-trait GWAS. When both MT-GWAS matched this condition, their CS were merged.

### Annotation of associated variants

In each CS, we searched for candidate causal variants. In addition to the statistical evidence, we relied on the annotation of the variants in the CS obtained with Variant Effect Predictor (VEP) v95.0 [51] that provides also the predicted impact (from MODIFIER to HIGH) and the SIFT score for missense variants. PhastCons conservation scores across 30 vertebrates [52] and GERP scores across 91 mammals [53] were used as conservation metrics. Furthermore, information available from the literature was considered for variants previously reported. Finally, we investigated whether SNPs in the CS overlapped with core and consensus segments called from ATAC-seq peaks in a recently released catalogue [54], or with CS of blood and liver cis-expression QTL (eQTL) reported in the same study. This eQTL study was selected as we had access to all the data. We also checked the overlap with eQTL from the cattle GTEx study [55]. To that end, we downloaded the tables from summary statistics and selected eQTL by application of a false-discovery rate of 0.05 (using the script provided in the original study).

### Conditional mapping

Subsequently, one candidate variant was selected per CS to perform a conditional mapping scan by fitting them as fixed effects in the LMM. For each QTLR, this conditional mapping allows to determine how well the tested candidate causal variant captures the QTL signal and whether it captures the signal for different traits, providing eventually further evidence for causality and pleiotropy. In addition, it allows to determine whether a single or multiple QTL affect the same trait in the QTLR. The conditional mapping was performed in 11 10-Mb regions centered around the lead variants and encompassing 516,465 SNPs (see Additional file 2: Table S3). Using the same approach as before, we estimated that the number of independent tests was approximately equal to 6000. For each QTLR, the conditional analyses were performed only for traits presenting evidence for association in the first GWAS (− log_10_P > 7), resulting in approximately 3.1 independent traits per region on average. As a result, we set the significant threshold to 2.5e−6 (− log_10_P > 5.6) to account for a total of approximately 20,000 independent tests. We repeated the same procedure if new significant QTL were detected in the QTLR.

## Results

### Identification of 11 QTL regions that affect multiple traits

**Fig. 1:**
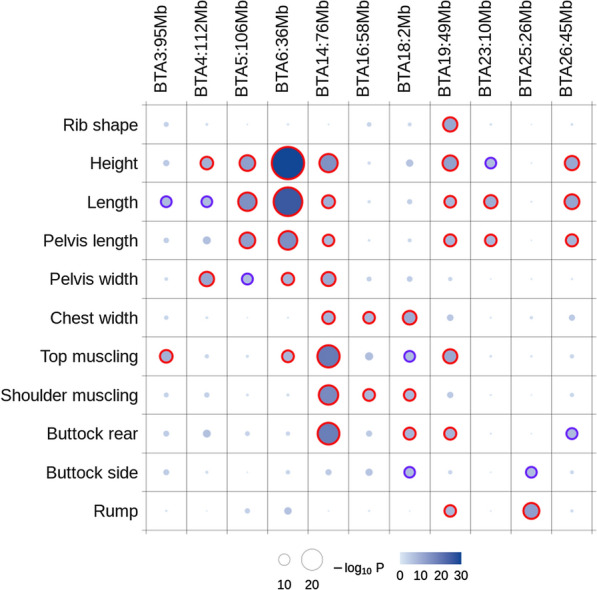
Summary of identified QTL regions (QTLR). QTLR are labelled according to the corresponding chromosome and the position (rounded in Mb). The maximum significance level for each trait per QTLR is indicated by the size and color intensity of the circles. Significant QTL are indicated with red outer circles, whereas dark purple outer circles are used for QTL reaching significance levels for a single genome scan

**Fig. 2:**
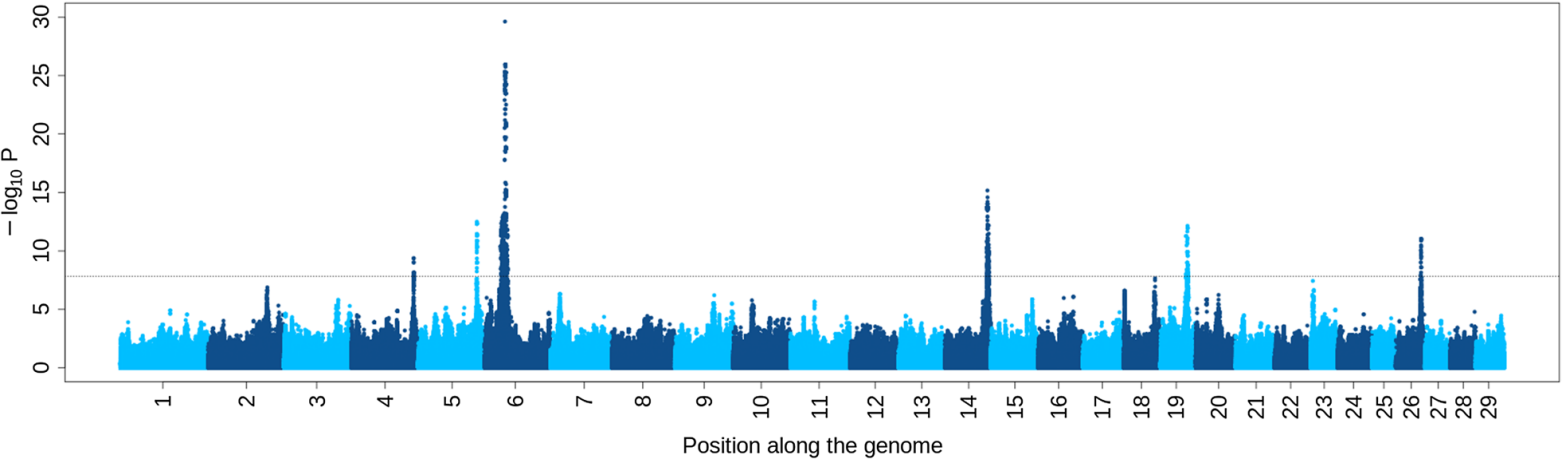
Manhattan plot for association with height. The horizontal line represents the significance level after correction for multiple testing

**Fig. 3:**
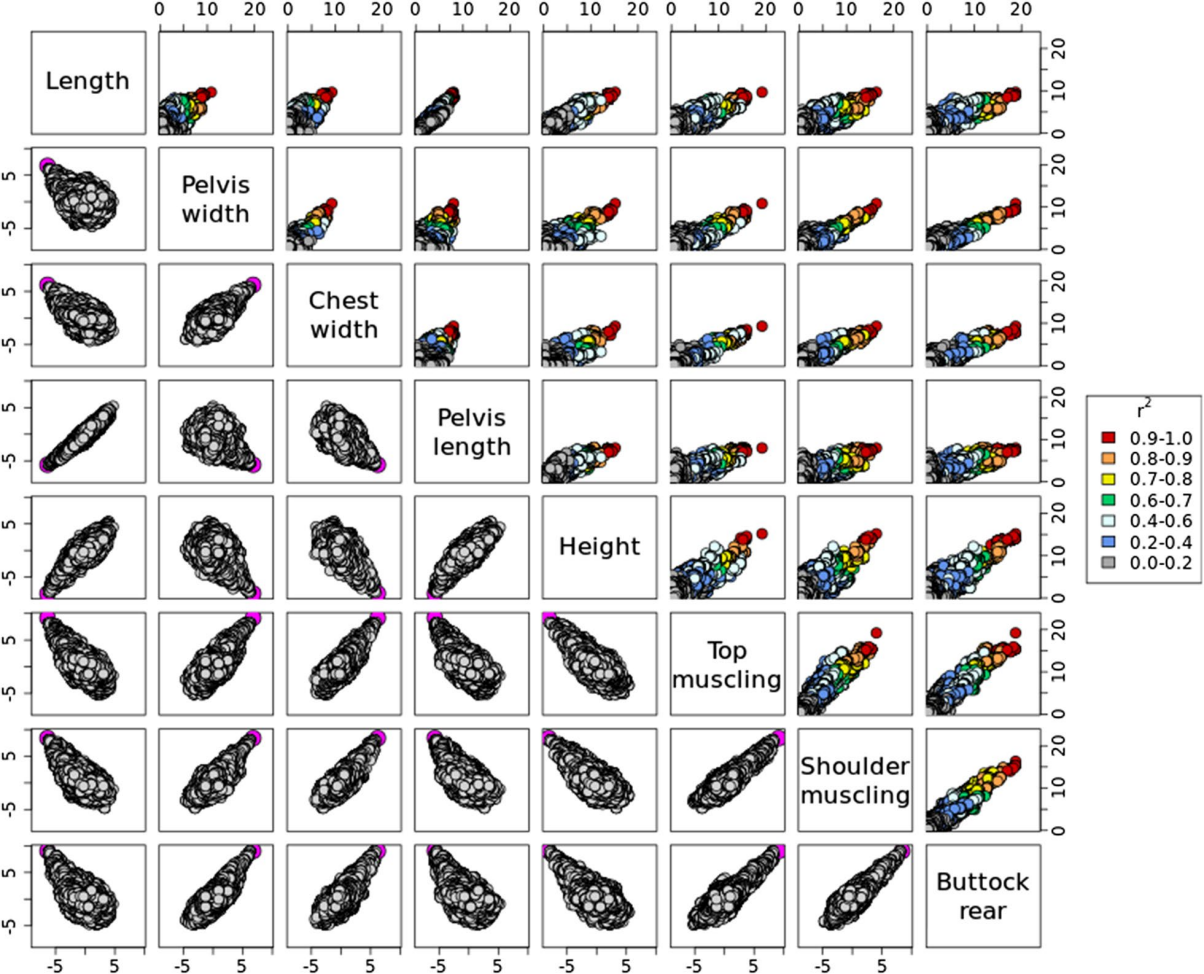
Scatterplots with association levels for different traits for the QTL region (QTLR) on BTA14. The selected traits are those harboring a significant signal in the QTLR. Upper diagonal: scatterplots with p-values on a negative log10 scale. The color represents the LD level with the lead SNP (from the trait with the strongest association). Lower diagonal: scatterplots with signed t-values. The magenta circle denotes the lead variant

Application of the ST-GWAS for 11 distinct traits resulted in the identification of 37 QTL (Fig. 1, and see Additional file 2: Table S4). The most significant QTL (p < 1e−15) were located on *Bos taurus* autosomes (BTA) 5, 6 and 14 and mainly associated with traits such as height or body length (Fig. 2). Based on their position, the QTL could be organized in 11 groups or QTLR (with a distance of less than 1 Mb between peaks). Most of the QTLR affected multiple traits (Fig. 1), with QTLR on BTA14 and BTA19 harboring significant associations with respectively eight and seven traits. In these QTLR, correlations between association levels or t-values obtained for different traits were often higher than 0.70 (see Additional file 3: Table S11). This is illustrated in Fig. 3 for the QTLR on BTA14 (see Additional file 1: Figs. S2–S9 for other traits). In general, effects for traits such as height or length were negatively correlated with effects estimated for muscular development traits. In addition, the credible sets (CS) of candidate variants identified for different traits overlapped for several QTLR. For instance, the IBSS-CS (obtained with SuSiE) from all the traits presenting a significant association in a QTLR shared at least one variant for eight of the QTLR (out of nine QTLR associated to two or more traits—see Additional file 2: Table S5). For three QTLR, the CS were even identical across all traits. Similar results were obtained when using LD-based CS (LDCS) obtained by selecting all SNPs with an r² > 0.80 with the lead variants (slightly fewer SNPs sharing with a threshold of r² > 0.90). Both approaches pointed to similar CS. For instance, LDCS (r² > 0.80) and IBSS-CS shared at least one common candidate variants for 34 out of 37 QTL, and the IBSS-CS was totally included in the LDCS for 29 QTL (see Additional file 2: Table S6). Overall, there is strong evidence that several of the QTLR harbor pleiotropic variants and thus, we decided to combine multiple-trait information to define MT LD-based CS. This MT mapping was performed in two groups of six traits related either to body size or to muscular development (see list in “Methods”). The resulting MT LD-based CS are described in Table 1. The median number of SNPs included in these MT LD-based CS was equal to 11 (ranging from 1 to 116; mean = 30), and their span ranged from 1 bp to 1.7 Mb (median = 268 kb). Only one or two associated genes (i.e., genes with coding, intergenic or up/down stream variants in the CS) were generally found in these CS (only two regions with more than three associated genes). Full details on ST and MT CS for the 11 QTLR are provided in Additional file 3: Tables S12–S22.

**Table 1:** LD-based credible sets (CS) identified by multiple-trait genome-wide association study (MT-GWAS) (r² > 0.90)

QTL-region	Number of SNPs in MT-GWAS1	Number of SNPs in MT-GWAS2	Number of SNPs in both CS	Span of CS in bp^a^	Number of genes	Genes present in CS
BTA3:95 Mb	2	2	2	1,733,382	2	SCP2^*^, RNF11^*^
BTA4:112 Mb	57	–	57	420,567	3	*ENSBTAG00000052473*, *CUL1*, *EZH2*
BTA5:106 Mb	6	–	6	6741	1	*CCND2*
BTA6:36 Mb	1	1	1	1	0	
BTA14:76 Mb	116	116	116	266,423	1	WWP1^*^
BTA16:58 Mb	11	11	11	251,954	1	PAPPA2^*^
BTA18:2 Mb	86	86	86	268,177	11	ENSBTAG00000052687^*^, ENSBTAG00000053632^*^, ENSBTAG00000051242^*^, IL34^*^, FUK^*^, ST3GAL2^*^, DDX19A^*^, DDX19B^*^, AARS^*^, EXOSC6^*^, CLEC18C^*^
BTA19:49 Mb	5	5	5	1,649,747	3	CDC27^*^, MRC2^*^, KCNH6^*^
BTA23:10 Mb	6	–	6	30,728	1	*ARMC12*
BTA25:26 Mb	–	20	20	647,161	18	*ENSBTAG00000048352*, *ENSBTAG00000051451*, *NUPR1*, *GDPD3*, *YPEL3*, *FAM57B*, *C16orf92*, *SEZ6L2*, *ASPHD1*, *MVP*, *SPN*, *CD2BP2*, *TBC1D10B*, *MYLPF*, *SEPT1*, *ZNF48*, *ZNF771*, *DCTPP1*
BTA26:45 Mb	4	14	18	474,978	2	*ENSBTAG00000007010*, *ADAM12*

*MT-GWAS1* MT-GWAS with traits related to height and body dimensions, *MT-GWAS2* MT-GWAS with traits related to muscular development

^*^Genes present in CS from both MT-GWAS are indicated with a star (other are specific to a single MT-GWAS)

^a^The start and end of each QTL region can be found in Additional file 3: Tables S12–S22 and S24–S28 that provide the detailed CS

### Four QTLR are associated to recessive deleterious coding variants

**Fig. 4:**
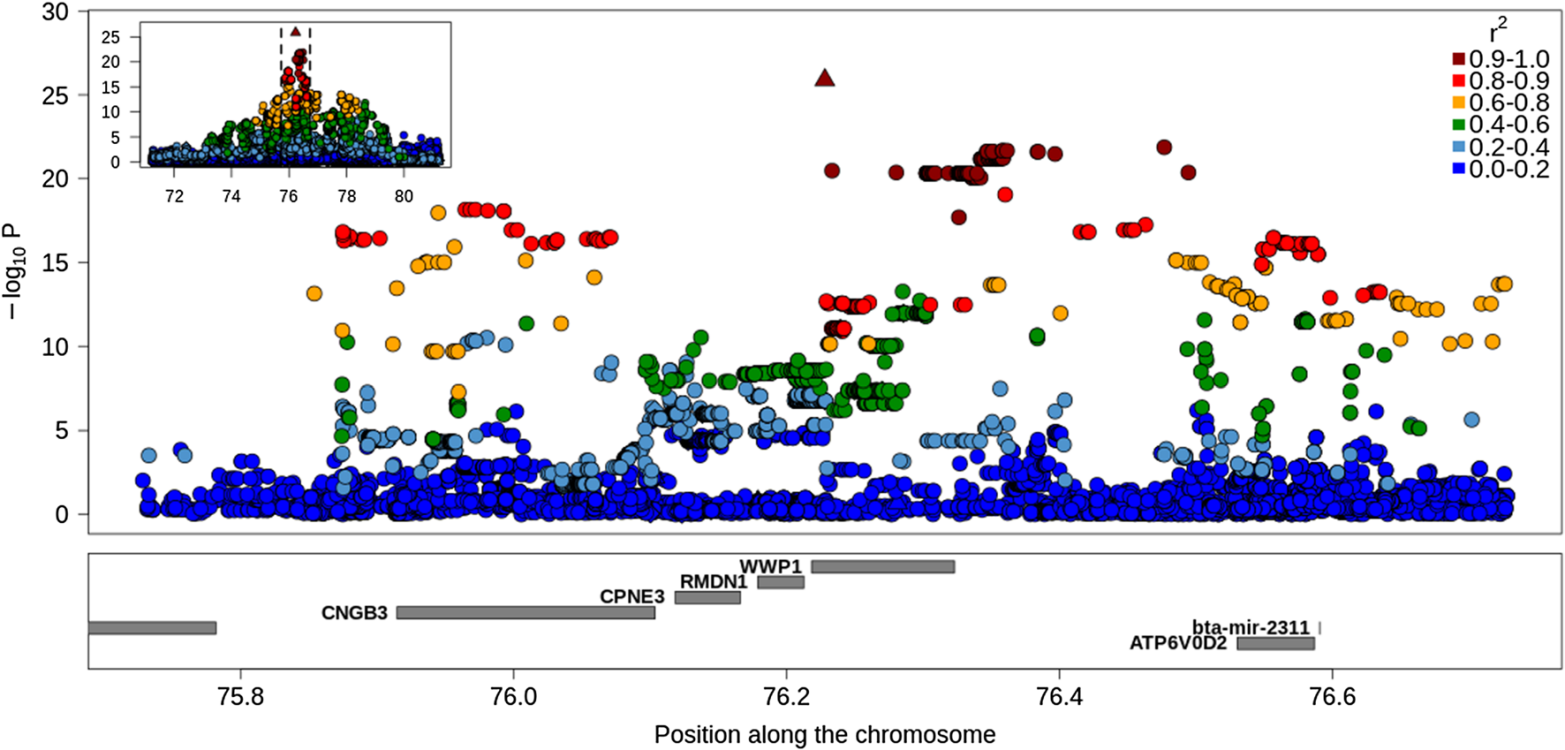
Regional association plot for the QTL region (QTLR) on BTA14. The results correspond to the MT-GWAS with traits related to body size. The colors represent the LD level with the lead variant and the symbols indicate the predicted impact of the variant (circle: modifier, diamond suit: low impact, up-pointing triangle: moderate impact, square: high impact). The positions of the genes are in the lower track

Four of the QTLR were associated to recessive deleterious variants previously identified in BBB cattle (see Table 2), three of which cause genetic defects when in the homozygous state [18, 19, 56]. These variants were indeed present in the MT LD-based CS (Fig. 4; and see Additional file 1: Fig. S10), and the LoF variant in *RNF11* associated to dwarfism, the 2 bp-deletion in *MRC2* associated to the crooked-tail syndrome (CTS) and the missense variant in *WWP1* reported in Charlier et al. [20] were even the lead SNP in both MT-GWAS (see Additional file 2: Table S7). The variants in *RNF11* and *WWP1* were also the lead variants in all but one of the ST GWAS. The variant in *MRC2* was the lead SNP in three out of seven ST GWAS and was included in three ST-LD based CS when the LD threshold was set to r² ≥ 0.90 (six if the threshold was relaxed to r² ≥ 0.80). Remarkably, the three variants were always present in the IBSS-CS and the variants associated with dwarfism and the CTS had always a PIP > 0.95 (i.e., the CS contained thus only this variant). The statistical evidence supporting these candidate variants is thus strong. Finally, the variant in the *ATP2A1* gene associated with congenital muscular dystrophy [56] presented an LD of r² = 0.88 with the lead SNP in both the ST GWAS for rump and the MT GWAS for muscular development traits (see Additional file 1: Fig. S10). It should be noted that this variant segregates now at low frequency in the population (*f* = 0.011) and achieves genome-wide significance only for one trait. Overall, the analysis of these previously identified variants shows that the MT LD-based approach is efficient and improves the resolution of the ST LD-based approach.

**Table 2:** Annotation of candidate or lead variants for the 11 QTL regions

BTA	Position	REF	ALT	Frequency	Gene	Consequences	SIFT score	GERP score	Phastcons
3	95,015,373	T	C	0.050	*RNF11*	Splice site variant		0.862	0.976
4	112,030,024	T	C	0.470	*EZH2*	Missense variant I549M	0.03	0.222	1
5	105,769,735	C	T	0.700	*CCND2*	Regulatory (ATAC-Seq, eQTL)		− 4.2	0
6	36,226,849	A	AT	0.050		Intergenic variant		0.078	–
14	76,227,910	C	T	0.140	*WWP1*	Missense variant R844Q	0.00	0.530	1
16	57,725,284	C	A	0.720	*PAPPA2*	Missense variant P282T	0.00	–	1
18	1,673,649	A	AT	0.270		Regulatory (ATAC-Seq)		0.149	–
19	47,095,175	CAG	C	0.030	*MRC2*	Frameshift variant		0.504	0
23	9,716,619	G	A	0.480	*ARMC12*	Regulatory (ATAC-Seq, eQTL)		− 0.678	–
25	25,933,247	G	A	0.010	*ATP2A1*	Missense variant R559C	0.00	0.222	0.984
26	45,553,105	G	A	0.590	*ADAM12*	Missense variant A582V	0.03	1.220	0.890

The frequency is reported for the alternate allele and SIFT scores are provided for missense variants

*BTA* *Bos taurus* chromosome, *REF* reference allele, *ALT* alternate reference

### Identification of three new missense variants in genes associated with growth disorders in other species

**Fig. 5:**
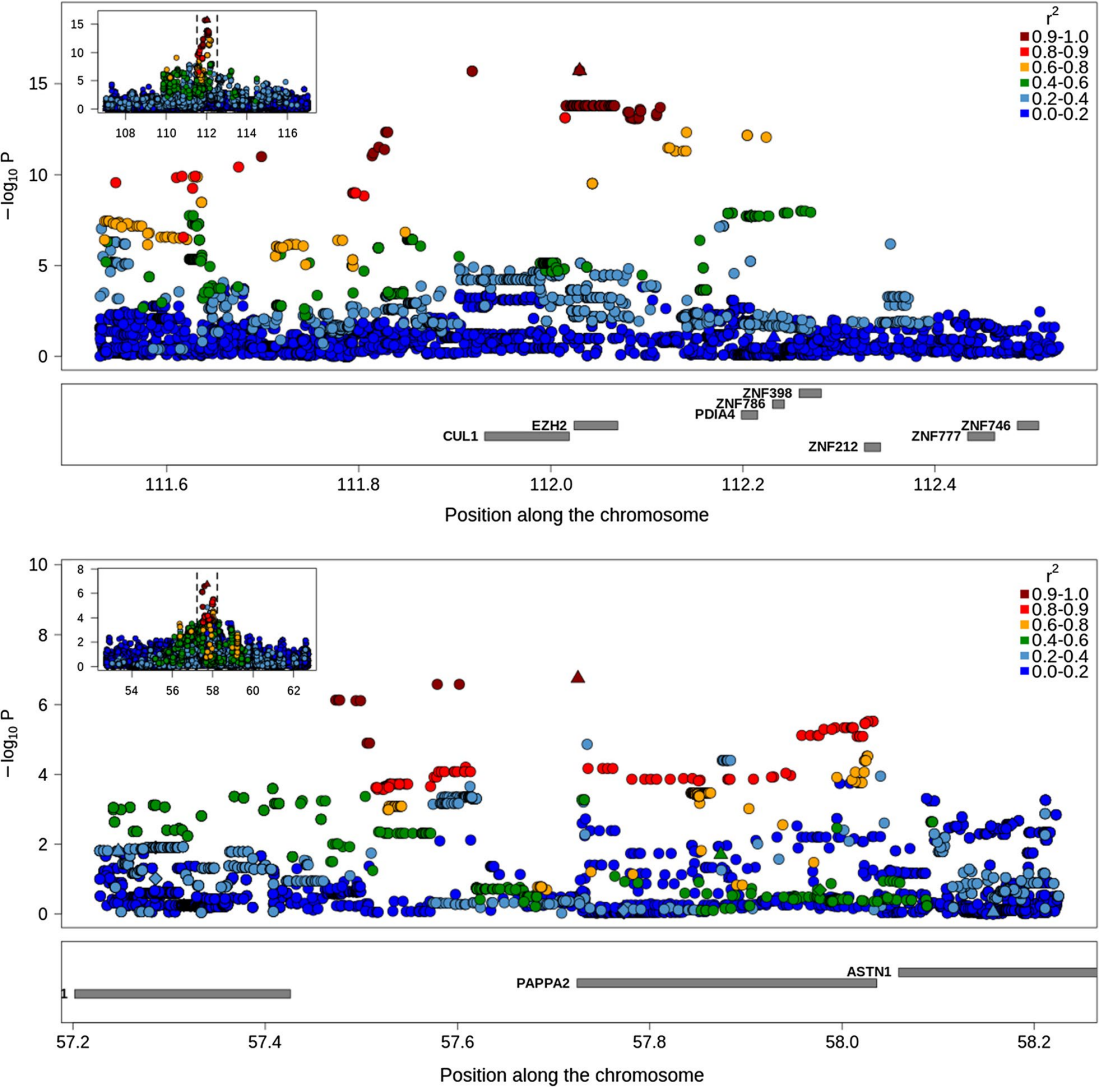
Regional association plot for the QTL regions (QTLR) with missense variants in *EZH2* and *PAPPA2*. The colors represent the LD level with the lead variant and the symbols indicate the predicted impact of the variant (circle: modifier, diamond suit: low impact, up-pointing triangle: moderate impact, square:  high impact). The positions of the genes are in the lower track. Upper panel: results of the MT-GWAS with traits related to body size on BTA4 and encompassing *EZH2*; lower panel: results of the MT-GWAS with traits related to muscular development on BTA16 and encompassing *PAPPA2*

For three additional QTLR, the MT LD-based CS contained non-synonymous variants in genes that have been previously associated with human height or growth (see “Discussion” for more details). These include a I549M missense variant in *EZH2* (QTLR on BTA4: Fig. 5), a P282T missense variant in *PAPPA2* (QTLR on BTA16; Fig. 5) and a A582V missense variant in *ADAM12* (QTLR on BTA26; see Additional file 1: Fig. S11). Note that these genes have multiple transcripts and thus the position of the amino acid change might vary (the genomic coordinates and alleles of the variants are available in Table 2). These three variants have strong statistical support (see Additional file 2: Table S7). The two first missense variants were indeed the lead SNP in the MT GWAS whereas the third was almost in perfect LD (r² = 0.998) with the lead SNP of the MT GWAS for muscular development traits (see Additional file 1: Fig. S11). In addition, each of these variants was also the lead SNP and present in both IBSS and LD-based CS for at least one ST GWAS.

In total, six coding variants were identified in the 11 MT LD-CS associated with six QTLR (when using an LD threshold of r² ≥ 0.90). Using the proportion of variants with a moderate or high predicted impact (mainly missense, splice site and frameshift variants and premature stop codons) in our data (0.34%) and the size of each CS, we estimated by random sampling (10^8^ repetitions) that we expect only 1.1 such variants on average in our CS (see Additional file 2: Table S8 for details on the enrichment analysis). The enrichment of these variants in our CS was significant (p = 0.001) and the number of CS harboring at least one coding variant was also significantly higher than expected (p = 2.0e−6). The chance to have a coding variant as lead variant for five or more CS was even lower (p < 1e−8). If we define CS using a r² ≥ 0.80 LD threshold, five additional coding variants would be identified including the variant in *ATP2A1* mentioned above and a frameshift variant in *LCORL* (a 2 bp deletion ACT>A at position 37,401,770) included in a long LD block with 73 variants spread over 2 Mb (see Additional file 1: Fig. S12). This would lead to a total of 11 coding variants located in eight distinct QTLR regions (MT LD-based CS containing then on average 73 variants). The number of observed coding variants and the number of CS harboring at least one coding variants would still be significant (p = 1.3e−4 and 3.1e−6).

### Evidence for regulatory variants among QTLR

For the QTLR without obvious candidate coding variants, we also investigated whether SNPs in the CS overlapped with core and consensus segments from ATAC-seq peaks present in the catalogue from Yuan et al. [54], with cis-eQTL reported in the same study or eQTL from the cattle GTEx study [55]. Contrary to the lead variant on BTA6, the CS variants on BTA5, consisting in six intronic variants from *CCND2* (see Additional file 1: Fig. S13), fall in consensus ATAC-seq peaks and even in the CS from an eQTL affecting *CCND2* expression in liver [54] (the lead SNP matches both criteria, the alternate allele being associated with increased expression and higher height). Another SNP from the CS affects expression of *TIGAR* in muscle (see Additional file 3: Table S23) [55]. Interestingly, a SNP located at position 105,773,809 (A>G) and in LD with the lead SNP (r² = 0.89) was the lead variant detected from a meta-analysis involving 18 breeds [8] and subsequently identified as a significant trans eQTL across multiple genes and tissues [57]. On BTA18, the CS contained 86 variants associated with 12 genes (see Additional file 1: Fig. S14). Several of these variants were located in both consensus and core ATAC-seq peaks. Finally, in the CS for the QTLR on BTA23 containing only six SNPs (see Additional file 1: Fig. S15), four intergenic variants match core ATAC-seq peaks and are in the CS from a blood eQTL reducing *ARMC12* expression levels [54]. The lead SNP located upstream from *ARMC12* is the lead SNP of this eQTL, the alternate allele is associated with lower expression and lower height. The regulatory effect of this locus is confirmed in the cattle GTEx data [55]. Indeed, two variants, including the lead SNP, are associated with *ARMC12* expression in blood, whereas two other variants regulate expression of *FKBP5* in muscle (see Additional file 3: Table S23).

### Stepwise conditional mapping: identification of multiple independent associations in *CCND2* and *LCORL* and of an additional deleterious coding variant

**Fig. 6:**
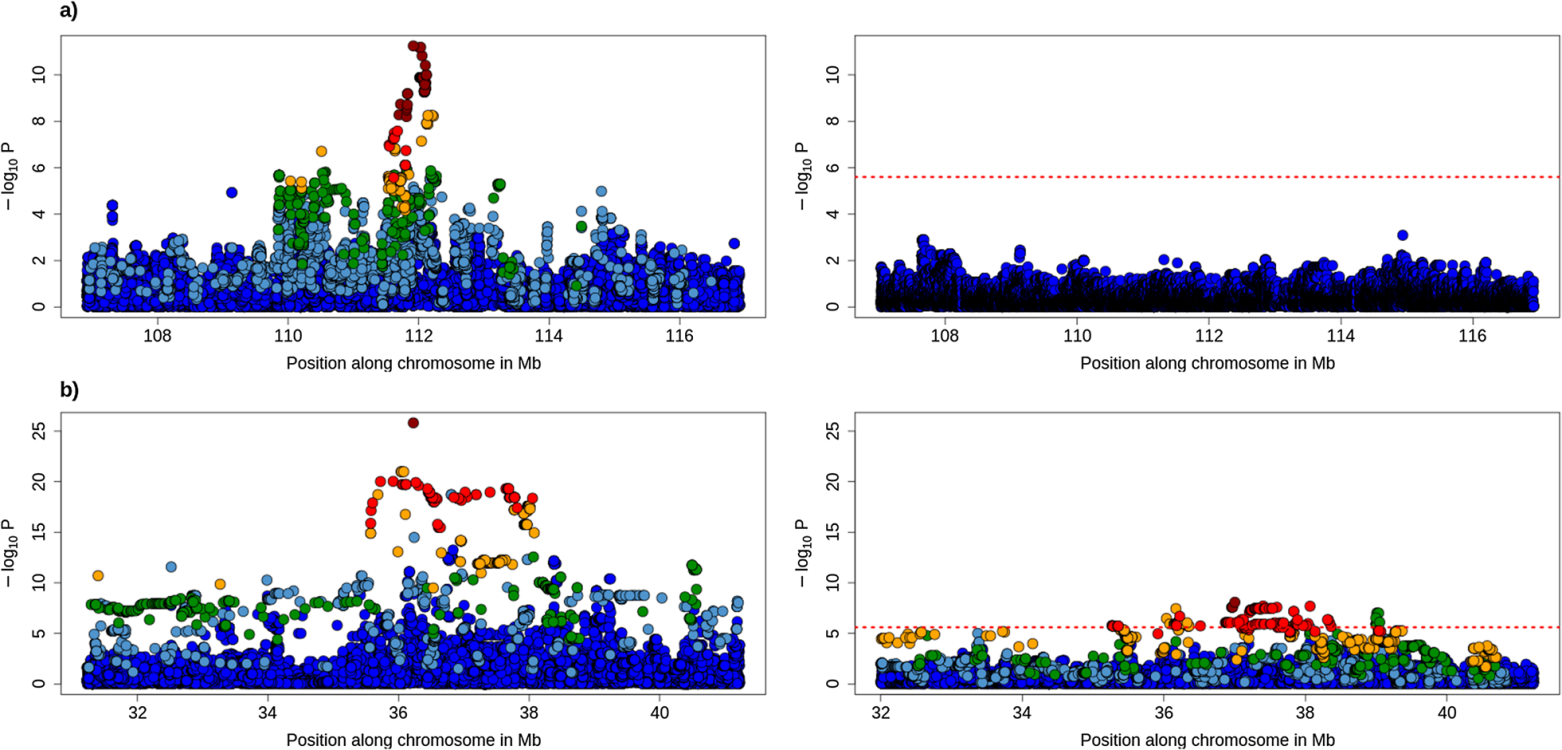
Regional association plot for the conditional mapping in the QTL regions (QTLR) on BTA4 and BTA6. The left panels represent the initial GWAS whereas the right panels correspond to conditional GWAS in which the candidate variants are fitted as covariate. The colors represent the LD level with the lead variant. The positions of the genes are in the lower track. **a** GWAS for pelvis width on BTA4, and **b** GWAS for length on BTA6

For each QTLR, we selected variants to fit as covariates in a secondary mapping analysis. In QTLR with candidate coding variants, we chose these as they were excellent functional candidates and presented very strong statistical significance (e.g., lead SNP in MT GWAS). For the other QTLR, we used the lead SNPs for subsequent analysis (Table 2). We performed the conditional mapping in 10-Mb regions centered around the selected variants. As for the initial mapping, details of CS are available in Additional file 3: Tables S24–S28. For the six QTLR on BTA3, 4, 14, 16, 18 and 26, no new significant associations were detected with the conditional mapping (Fig. 6, and see Additional file 1: Fig. S16, and Additional file 2: Table S9), indicating that the fitted variant captured the QTL signal for all associated traits. For many of the QTL or QTLR, the signal dropped strongly (see for instance examples on BTA3, 4, 14 or 26). However, for the two QTLR regions mainly associated with body dimension traits and located on BTA5 and 6, new significant associations with the same group of traits were detected (Fig. 6, and see Additional file 1: Fig. S17). These QTLR presented among the most significant associations in the first scan, and still harbor highly significant associations (p < 1e−7 and 1e−8, respectively). On BTA5, the exact same group of three traits was associated (size, body length, pelvis length) and all the CS encompass a single SNP downstream of *CCND2*. On BTA6, the association was significant for size and body length. For the initial mapping, the lead SNP from the MT GWAS was an intergenic variant but the *LCORL* and *NCAPG* genes were located in the same region (see Additional file 1: Fig. S12). For the conditional mapping, the MT LD-based CS was particularly large, including several variants in *NCAPG* or *LCORL* embedded in a long haplotype block (Fig. 6). Among these, the variant with the largest predicted impact was a missense variant Y551C in *LCORL*, presenting an r² = 0.95 with the lead SNP. The LD between the lead SNP from the primary and secondary associations were low, respectively r² = 0.16 and r² = 0.01 for QTLR on BTA5 and 6, indicating independent associations. Thus, for both these QTLR, the conditional mapping identifies a second independent QTL associated with the same set of traits and pointing to the same genes.

For the three last QTLR regions, significant associations were still present but at lower magnitude (p > 1e−7). These signals would not be significant for a whole-genome scan but indicate that all the primary signal has not been fully captured by fitting our candidate variants. For the QTLR on BTA19, the most significant signals drop after inclusion of the LoF variant in *MRC2* (see Additional file 1: Fig. S18). However, associations are significant for body length and top muscling (− log_10_P > 5.6) whereas there is still some evidence for association with size or rump (p < 1e−5). For body length and top muscling, the CS contains a single SNP (respectively, an intronic variant in *MAPT* and an intergenic variant). These four associations point to four different regions, indicating a quite complex QTLR. On BTA23, the lead variant, associated with *ARMC12*, captured the signal for body length and pelvis length whereas for height, there was a signal for a second QTL (p = 2.4e−6) located at more than 2 Mb (see Additional file 1: Fig. S17). The lead SNP was an intronic variant in *BOLA-DOB* (the CS contained only one more SNP). It should be noted that, as for the HLA region in humans [58], this is a complex region with high nucleotide diversity levels and characterized by the presence of copy number variations [59]. As a result, LD levels and imputation accuracy are reduced. Finally, for the QTLR on BTA25, there was no longer evidence for association with rump after inclusion of the variant in *ATP2A1* in the model (see Additional file 1: Fig. S19). However, this variant did not capture the signal associated with muscular development of the buttock (side view) for which the association was still strong (p = 1.35e−7; see Additional file 1: Fig. S19). Thus, there is evidence for two linked QTL that affect two distinct traits in that QTLR. Two distinct and distant peaks achieved similar significance levels (see Additional file 1: Fig. S19; Additional file 3: Table S28), the CS for the first peak consisted in a single intergenic variant whereas the second CS contained 15 SNPs (r² > 0.90). An R631W missense variant in *ATP2A1* previously shown to negatively affect meat quality and muscular development [60] was in high LD (r² = 0.88) with the lead variant (see Additional file 1: Fig. S20) and represents thus the best candidate causative variant.

We repeated a conditional mapping by adding the lead variants for the secondary QTL on BTA5, 6, 23 and 25. For all tested traits and QTLR, we did not detect new significant associations after correction for multiple testing (see Additional file 2: Table S10). The p-values were indeed higher than 1e−4, whereas the significance threshold was set to 2e−5 (considering ~ 2500 independent SNPs in the four tested QTLR).

Additional file 3: Tables S29 and S30 provide the effect sizes of candidate or lead variants for all traits and the proportion of genetic variance they account for (estimated in a model where all the variants are fitted simultaneously). Significant alleles account generally for 1 to 2% of the genetic variance, and up to 5% for the variant on BTA6. Together the 15 variants capture around 20% of the genetic variance for traits related to height and length, and from 6 to 11% for muscular development traits. These values were in agreement with the relative reduction of polygenic variance when these variants were fitted in the model (see Additional file 3: Table S30). These values might however be overestimated and must be confirmed in an independent dataset.

## Discussion

### Identification of candidate causal coding variants for the majority of QTLR

In this study, we performed a sequenced-based association study for 11 traits related to muscular development and body dimension in a cohort of ~ 15,000 BBB cattle cows. We identified 11 QTLR with genome-wide significant associations and most of them affected several correlated traits. Several coding variants included in our CS represented strong candidate causal variants. Five of these correspond to deleterious variants specific to BBB that have been previously characterized [18–20, 56, 60]. In addition, we found three new missense variants in *PAPPA2*, *ADAM12* and *EZH2*, three genes related to growth disorders in different species including humans. Indeed, the role of *PAPPA2* on growth has been documented in multiple species, it is a regulator of *IGF1* and is associated with short stature in humans [61, 62] and in mice [63, 64]. *ADAM12* was identified as a susceptibility gene for the Kashin-Beck disease in humans, causing growth retardation [65]. In agreement, *ADAM12*-deficient zebrafish present growth retardation [66]. In humans, mutations in *EZH2* cause the Weaver syndrome and increased height [67], tall stature [68] but also growth retardation and severe short stature [69]. Two of these coding variants were the lead variants in their respective MT-GWAS. Thus, they are strong candidates as they have strong statistical support, they change the protein sequence, and coding variants in the same genes are known to affect growth in other species. The three variants are predicted to be deleterious (SIFT score < 0.05) and have high PhastCons scores (> 0.88) and positive, although not extreme, GERP scores (from 0.22 to 1.22). In addition, two independent signals on BTA6 might be associated to a missense and a frameshift variant in *LCORL*, a gene associated with height in different cattle breeds [8] and several species [7, 11–13]. To our knowledge, these are the first coding variants in *LCORL* that are significantly associated with height reported in cattle. The Y551C missense variant in *LCORL* was predicted to be tolerated (SIFT score = 0.46) and was not conserved (0.00 PhastCon; − 2.37 GERP score). In both cases, the variants were included in a long haplotype block encompassing many variants making it more difficult to pinpoint the causal variant. In addition, the frameshift variant was not in very high LD with the lead SNP (r² = 0.84). The evidence for their causality is thus weaker, although they might affect the protein function of a strong candidate gene. Overall, the number of coding variants in our CS and the number of CS harboring at least one coding variant were significantly larger than expected by chance (see Additional file 2: Table S8). These enrichments suggest that a fraction of these coding variants are causal, in particular if we consider that several of them were lead SNPs (which is even less likely by chance) and that they fall in genes previously associated with height. The number of QTL is too limited to make strong assumptions on the relative contribution of coding versus regulatory variants to genetic variation of complex traits. Our QTL represent only a fraction of the variants contributing to genetic variation, and correspond only to the largest effects segregating in the BBB cattle population (see Additional file 3: Tables S29, S30). Nevertheless, contribution of coding variants should not be underestimated.

### Evidence for association with regulatory variants

Beside these candidate coding variants, we found evidence for regulatory variants in three QTLR on BTA5, 18 and 23. CS from these three QTLR did overlap with the catalogue of regulatory regions identified by ATAC-SEQ by Yuan et al. [54]. For QTLR on BTA5 and 23, there was also association between the CS with cis-eQTL from a study conducted in blood and liver in Holstein [54] and evidence for regulatory effects in the cattle GTEx dataset [55]. In addition, the BTA5 QTL CS contained a SNP that was previously proposed as a candidate variant for a stature QTL and that significantly affects expression as a trans-eQTL in multiple tissues [55]. This illustrates how such catalogues can help to better understand mechanisms underlying identified QTL. Ideally, catalogues of eQTL obtained from experiments in the most relevant tissues from individuals from the same breed should be used. Such data was not available for the present study and future experiments might improve the annotation.

### Candidate causal variants in genes that affect stature in multiple species and breeds

In 2011, Pryce et al. [6] concluded that genes associated with height in humans also control stature in cattle. In agreement, Bouwman et al. [8] demonstrated that genes associated with height in cattle GWAS were enriched in genes also reported in human GWAS for the same trait. Raymond et al. [70] identified such shared genes by comparing associations found in humans by Wood et al. [71] or by Yengo et al. [72] with those found in cattle by Bouwman et al. [8]. In our study, several candidate variants were also associated to genes previously associated with growth or height in cattle and in other species. First, the most significant QTLR located on BTA6, included *LCORL* and *NCPAG*, that have been linked with height in cattle based both on association studies [8, 73] and signatures of selection [8, 13]. Similar findings have been reported in other species including humans, dog and horse [11, 12, 74]. In cattle, associations have been observed in several breeds [8]. Second, the region on BTA5 was among the most significant regions and the CS included only one gene, *CCND2*. This gene has also been previously associated with height in other cattle breeds [8, 75–77], and in other species such as humans [78, 79]. As in our study, the allele reported in humans by Stenthorsdottir et al. [78] was regulatory (increasing both expression and height). For both QTLR on BTA5 and 6, we identified two independent signals stressing the importance of these genes and strengthening the causality of *CCND2* (it was twice the single gene present in the CS). Next, *ADAM12* and *PAPPA2* are both associated with growth disorders (see above) and have been identified as ‘shared’ genes by Raymond et al. [70]. *PAPPA*, a paralog of *PAPPA2*, has also been listed by Pryce et al. [6] as a gene affecting height in both humans and cattle, and was proposed as candidate gene for size in horse by Petersen et al. [12]. Interestingly, the lead or candidate variants associated with *CCND2*, *LCORL*, *ADAM12* and *PAPPA2* are segregating in other breeds from the Run 3.0 of 1000 Bull Genomes Project [80] indicating that these variants are relatively old (see Additional file 3: Table S31). Overall, these results confirm previous findings, which indicate that a set of shared genes contribute to genetic variation of height in mammals. We strengthened the evidence that these genes are causal in cattle based on association results (e.g., lead variants, limited number of genes in the CS, multiple independent associations for some genes) and by the identification of coding variants in *PAPPA2* and *ADAM12*. Such candidate coding variants with strong statistical support (e.g., present as lead SNP for at least one GWAS) were not previously reported among the associations in cattle. Thus, *CCND2*, *LCORL*, *ADAM12* and *PAPPA2* appear to be associated with height in multiple breeds or species. In addition, the four genes present multiple associations in the human GWAS catalogues [81]: respectively 80, 26, 14 and 35 associations for *LCORL*, *CCND2*, *ADAM12* and *PAPPA2*. Similarly, associations with *LCORL* and *CCND2* and height (or related traits) are also reported in the Animal QTLdb (release 51) [82]. From these elements, we can thus conclude that these genes play an important role in genetic variation for height in multiple species.

### Candidate genes for other QTLR include genes associated with growth disorder and epigenetic regulation

For other QTLR, the candidate genes presented limited evidence for sharing across multiple species. For instance, *EZH2* is not associated with height in the cited GWAS catalogue, whereas *ARMC12* or the region on BTA18 encompassing genes such as *IL34*, *COG4*, *FUK*, *ST3GAL2*, *DDX19A* and *DDX19B*, present only associations in the largest cohort studies like those from Yengo et al. [83] or from Kichaev et al. [84]. In both studies, several genes are found in the associated genomic segments and the causal genes remain to be determined. Associations between height or muscular development and candidate genes from the three regions are also not reported in the Animal QTLdb (release 51) [82]. As mentioned above, *EZH2* is nevertheless associated to growth disorders. Interestingly, *ARMC12* increases the activity of *EZH2* [85] making a connection between both candidate genes. We did not find evidence for interactions among the two identified variants (i.e., the effect of the variant in *EZH2* is the same when estimated conditionally on the three possible genotypes at the *ARMC12* variant). These genes are involved in the polycomp repressive complex 2 (PRC2) that repress gene transcription during development through methylation [86]. *EZH2* encodes the histone methyltransferase of PRC2 [87, 88], whereas *ARMC12* facilitates the formation and activity of PRC2 [85]. These variants might thus play a role through epigenetic regulation. Unlike other identified genes, *EZH2* has not been reported in other GWAS for height. Interestingly, the missense variant is breed specific. Thus, variants in this pathway seem to contribute less often to variation in height.

There was no obvious candidate gene in the CS on BTA18 but we found evidence that regulatory variants might underlie this QTLR. Interestingly, the orthologous region in humans harbors enhancers. Such regulatory variants could influence other genes that overlap the QTLR. Among these, *COG4* is a potential candidate gene since it is the causal gene for the Saul-Wilson syndrome causing dwarfism and skeletal abnormalities in humans [89, 90], and is associated with reduced body length in zebrafish [91]. A mutant that affects skeleton and bone mineral density in mouse has been described in the Mouse Genome Informatics database [92]. Mutations in *COG4* have been shown to disturb the Wnt signaling pathway that plays an important regulation role during embryonic development [91].

### Breed-specific recessive deleterious variants are associated with height and muscular development traits

The four remaining QTLR were associated with five recessive deleterious variants previously identified in BBB cattle [18–20, 56, 60], including genetic defects [18, 19, 56]. These variants also present a selective advantage, resulting in an heterozygote advantage for most of them [18–20, 56, 60]. They are breed specific (i.e., not observed in other breeds from the Run 3.0 of 1000 Bull Genomes Project [35]; see Additional file 3: Table S31) and the associated genes, *RNF11*, *WWP1*, *MRC2* and *ATP2A1* are not unambiguously associated to height in the GWAS catalogue or in the Animal QTLdb (release 51) [82]. The observation of five deleterious variants that have a negative impact on fitness but contribute to variation in height or muscular development is rather unique, even if other deleterious variants presenting a heterozygote advantage have been reported in other livestock species [93–95]. The reason why such variants are regularly observed in BBB remains to be determined. However, it is tempting to speculate that the past and ongoing intensive selection for muscular development might play a role. The breed has indeed been driven far from an optimal phenotype in terms of fitness; slight additional improvements of muscular development might still be allowed but beyond a certain point selection could have negative consequences. For instance, as a result of the fixation of an 11-bp deletion in *MSTN*, BBB individuals can be considered as knock-out for this gene, a member of the transforming growth factor β (TGFβ) superfamily [96]. Consequently, *MSTN* is no longer playing its role as a negative regulator of skeletal muscle mass and such individuals present increased muscle mass (the so-called double-muscling phenotype) [96]. The genetic background where new mutations arise, and the potential impact of these new mutations might therefore be very different from those in other breeds that have functional *MSTN* alleles. This might impact the behavior of other members from the TGFβ family, and the consequences of their mutations. Interestingly, *RNF11* and *WWP1*, that each harbor one of the recessive deleterious variants presenting a heterozygote advantage, are such genes that regulate the TGFβ pathway [97], suggesting that other members of the family might indeed be impacted. Further investigations are nevertheless required to understand how intensive selection increases the number of such deleterious variants with a heterozygote advantage.

## Conclusions

We have performed a sequenced-based association study for traits related to muscular development and body dimensions in BBB cattle. We identified variants associated with height in genes that affect stature in multiple species and breeds, indicating a shared architecture among mammals. Some of these variants were old and present in several breeds. In addition, breed-specific variants were also identified. In particular, several recessive deleterious variants were significantly associated with height or muscular development. Their segregation in the breed might result from the extreme selection for muscular development. Overall, the BBB cattle represent an interesting model to study height and to identify new variants or new genes such as *EZH2* that underlie this trait.

### Supplementary Information

**Additional file 1: Figure S1.** Graphical summary of the analytical framework. **Figure S2.** Scatterplots for association levels for different traits for the QTL region on BTA4. **Figure S3.** Scatterplots for association levels for different traits for the QTL region on BTA5. **Figure S4.** Scatterplots for association levels for different traits for the QTL region on BTA6. **Figure S5.** Scatterplots for association levels for different traits for the QTL region on BTA16. **Figure S6.** Scatterplots for association levels for different traits for the QTL region on BTA18. **Figure S7.** Scatterplots for association levels for different traits for the QTL region on BTA19. **Figure S8.** Scatterplots for association levels for different traits for the QTL region on BTA23. **Figure S9.** Scatterplots for association levels for different traits for the QTL region on BTA26. **Figure S10.** Regional association plot for the QTLR associated to recessive genetic defects on BTA3, BTA19 and BTA25. **Figure S11.** Regional association plot for the QTLR on BTA26. **Figure S12.** Regional association plot for the QTLR on BTA6. **Figure S13.** Regional association plot for the QTLR on BTA5. **Figure S14.** Regional association plot for the QTLR on BTA18.**Figure S15.** Regional association plot for the QTLR on BTA23. **Figure S16.** Regional association plot for the conditional mapping in QTLR on BTA3, BTA14, BTA16 and BTA26. **Figure S17.** Regional association plot for the conditional mapping in QTLR on BTA5 and BTA23. **Figure S18.** Regional association plot for the conditional mapping in QTLR on BTA19. **Figure S19.** Regional association plot for the conditional mapping in QTLR on BTA25. **Figure S20.** Regional association plot for the conditional mapping in QTLR for the first peak on BTA25.

**Additional file 2: Table S1.** Summary statistics for linear classifications traits in Belgian Blue Beef cattle. **Table S2.** Number of individuals genotyped on the different SNP genotyping arrays. **Table S3.** Description of regions included in the conditional mapping analyses. **Table S4.** Description of the 37 identified QTL. **Table S5.** Information on credible sets: number of traits sharing at least one variant in their credible set and number of traits with identical credible sets. **Table S6.** Comparison of LD-based credible sets (CS) and CS obtained with the IBSS approach implemented in SuSiE. **Table S7.** Candidate or lead variants for the 11 QTLR. **Table S8.** Information used for the enrichment analysis. **Table S9.** Most significant associations levels achieved for each trait in the conditional mapping. **Table S10.** Most significant associations levels achieved for traits in the second iteration of conditional mapping.

**Additional file 3: Tables S11–S31.** Correlations and Credible sets. **Table S11.** Correlations among association levels in the QTL regions. **Table S12–S22.** Credible sets obtained with single and multiple trait approaches for the 11 QTL regions. **Table S23.** Variants from credible sets detected as eQTL in the Cattle GTEx study. **Table S24–S28.** Credible sets obtained with single and multiple trait approaches for the conditional mapping. **Table S29.** Effects of candidate variants estimated jointly. **Table S30.** Percentage of genetic variance associated with identified candidate variants. **Table S31.** Frequency of alleles of the candidate variants in other breeds.

## Data Availability

The data that support the findings of this study are available from Elevéo and Inovéo (Awé Group, Belgium) but restrictions apply to the availability of these data, which were used under license for the current study, and so are not publicly available.
