# Supplementary material for: Sequenced-based GWAS for linear classification traits in Belgian Blue beef cattle reveals new coding variants in genes regulating body size in mammals
Source: Genet Sel Evol. 2023 Nov 28;55:83. doi: 10.1186/s12711-023-00857-4 (PMC10683324; doi:10.1186/s12711-023-00857-4)
Supplement: Supplementary file 1 — Additional file 1: Figure S1. Graphical summary of the analytical framework. Figure S2. Scatterplots for association levels for different traits for the QTL region on BTA4. Figure S3. Scatterplots for association levels for different traits for the QTL region on BTA5. Figure S4. Scatterplots for association levels for different traits for the QTL region on BTA6. Figure S5. Scatterplots for association levels for different traits for the QTL region on BTA16. Figure S6. Scatterplots for association levels for different traits for the QTL region on BTA18. Figure S7. Scatterplots for association levels for different traits for the QTL region on BTA19. Figure S8. Scatterplots for association levels for different traits for the QTL region on BTA23. Figure S9. Scatterplots for association levels for different traits for the QTL region on BTA26. Figure S10. Regional association plot for the QTLR associated to recessive genetic defects on BTA3, BTA19 and BTA25. Figure S11. Regional association plot for the QTLR on BTA26. Figure S12. Regional association plot for the QTLR on BTA6. Figure S13. Regional association plot for the QTLR on BTA5. Figure S14. Regional association plot for the QTLR on BTA18.Figure S15. Regional association plot for the QTLR on BTA23. Figure S16. Regional association plot for the conditional mapping in QTLR on BTA3, BTA14, BTA16 and BTA26. Figure S17. Regional association plot for the conditional mapping in QTLR on BTA5 and BTA23. Figure S18. Regional association plot for the conditional mapping in QTLR on BTA19. Figure S19. Regional association plot for the conditional mapping in QTLR on BTA25. Figure S20. Regional association plot for the conditional mapping in QTLR for the first peak on BTA25. [file 12711_2023_857_MOESM1_ESM.pdf]

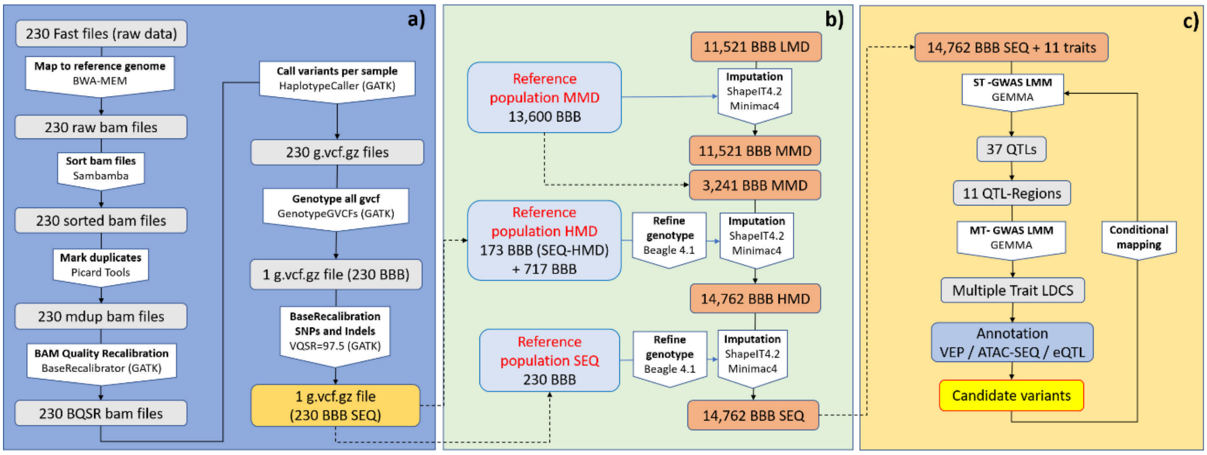

Figure S1. Summary of the analytical framework. **a)** Bioinformatic pipeline for whole-genome sequence data analysis and variant calling for 230 Belgian Blue beef (BBB) sires. **b)** Steps for genotype imputation from low marker density (LMD) marker arrays to whole-genome sequence (SEQ) level for the 14,762 BBB cows from the mapping population (11,521 genotyped on LMD and 3,241 genotyped on medium marker density (MMD) arrays). The genotypes from individuals from the high marker density (HMD) and the SEQ reference panels were refined using Beagle 4.1 whereas phasing was performed with ShapeIT4.2 and imputation with Minimac4. **c)** Genome-wide association studies (GWAS) were conducted with GEMMA on imputed whole-genome sequence data for 11 traits related to muscular development and body dimension, leading to the identification of 37 Quantitative Trait Loci (QTLs) corresponding to 11 distinct QTL regions (QTLR). Multiple-Trait GWAS (MT-GWAS) was run in the 11 QTLR to fine-map QTLs (two groups of 6 traits were defined, corresponding respectively to body dimension and muscular development traits). Credible Sets (CS) of candidate variants were annotated with VEP and using information from existing ATAC-SEQ and eQTL catalogues to identify candidate causative variants (CS from ST-GWAS were used as complementary information). In order to find independent association in the QTLR, a conditional GWAS was then performed using the selected candidate variants as covariates in the model.

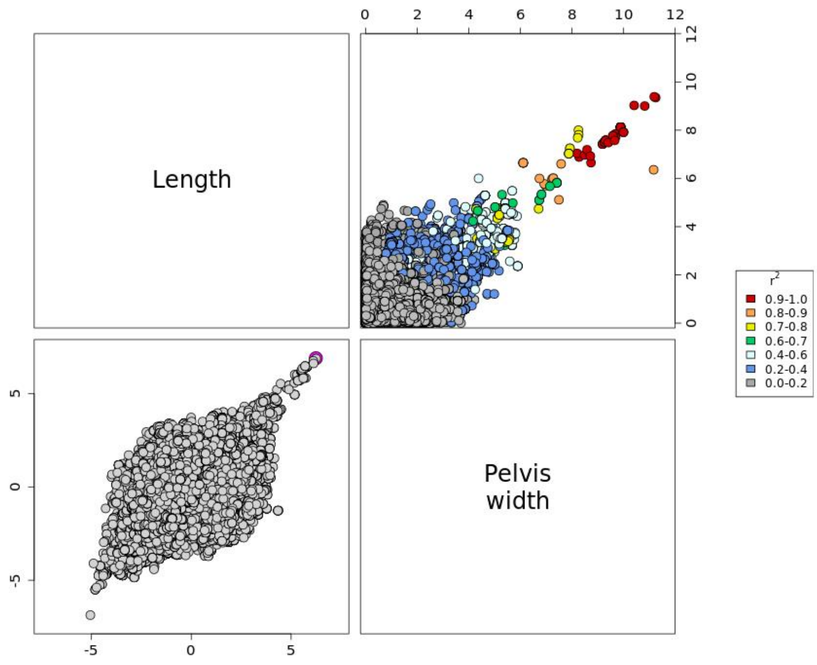

Figure S2. Scatterplots for association levels for different traits for the QTL region on BTA4. The selected traits are those harboring a significant signal in the QTLR. Upper diagonal: scatterplots with p-values on a negative log10 scale. The color represents the LD level with the lead SNP (from the trait with the strongest association). Lower diagonal: scatterplots with signed t-values. The magenta circle denotes the lead variant.

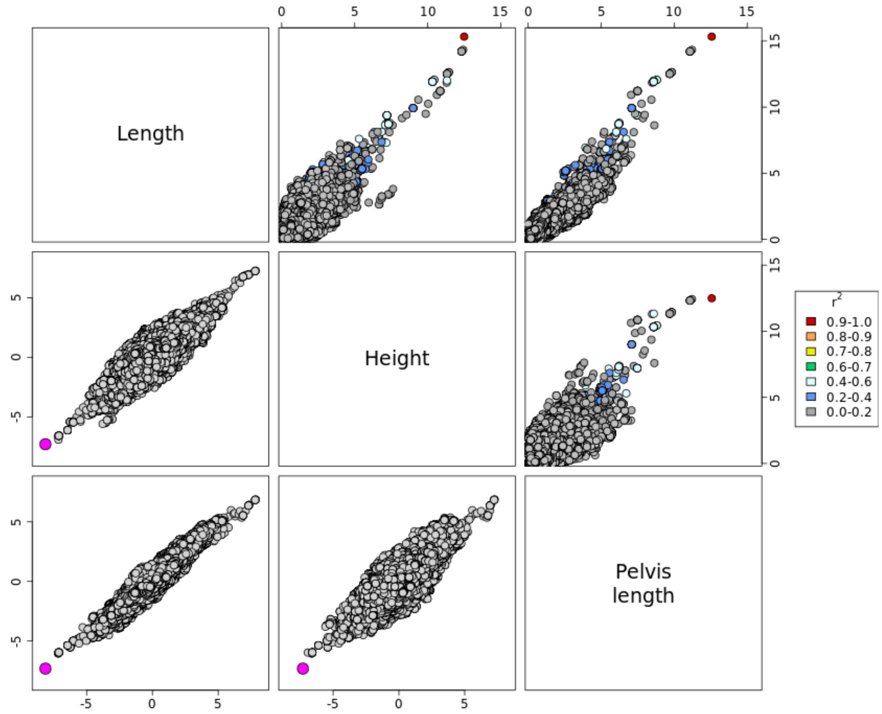

Figure S3. Scatterplots for association levels for different traits for the QTL region on BTA5. The selected traits are those harboring a significant signal in the QTLR. Upper diagonal: scatterplots with p-values on a negative log10 scale. The color represents the LD level with the lead SNP (from the trait with the strongest association). Lower diagonal: scatterplots with signed t-values. The magenta circle denotes the lead variant.

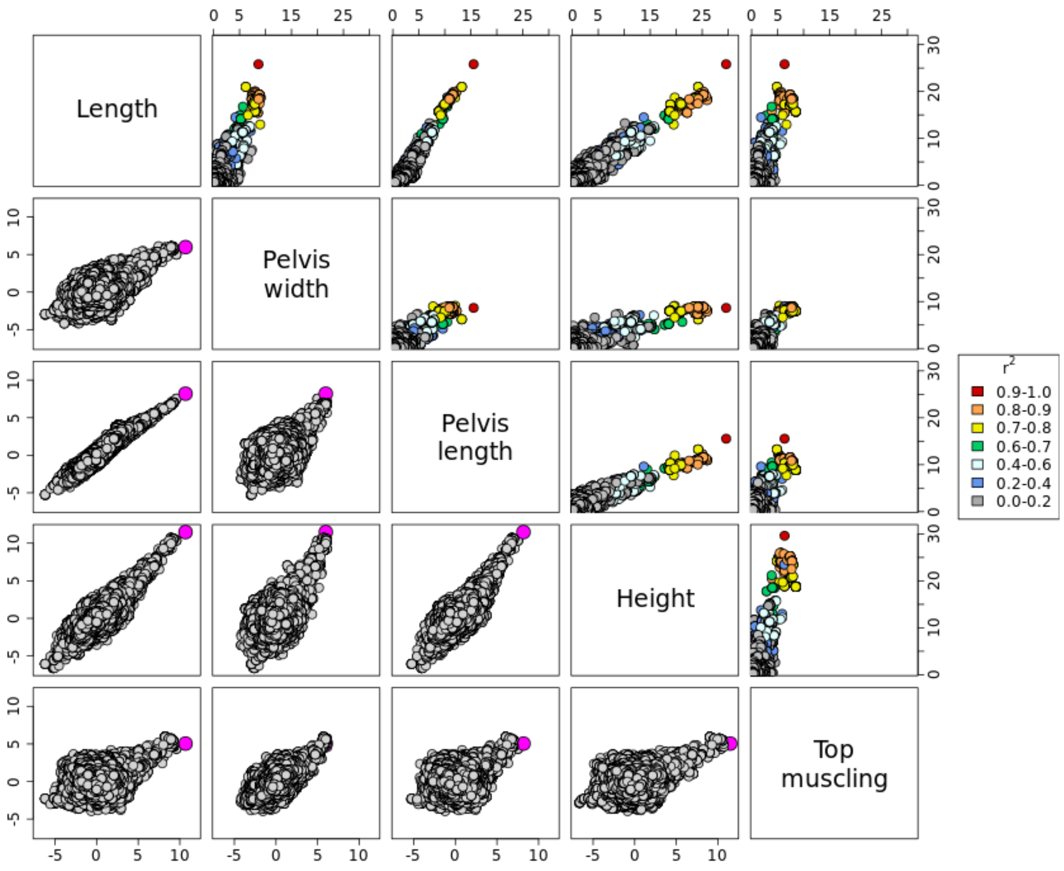

Figure S4. Scatterplots for association levels for different traits for the QTL region on BTA6. The selected traits are those harboring a significant signal in the QTLR. Upper diagonal: scatterplots with p-values on a negative log10 scale. The color represents the LD level with the lead SNP (from the trait with the strongest association). Lower diagonal: scatterplots with signed t-values. The magenta circle denotes the lead variant.

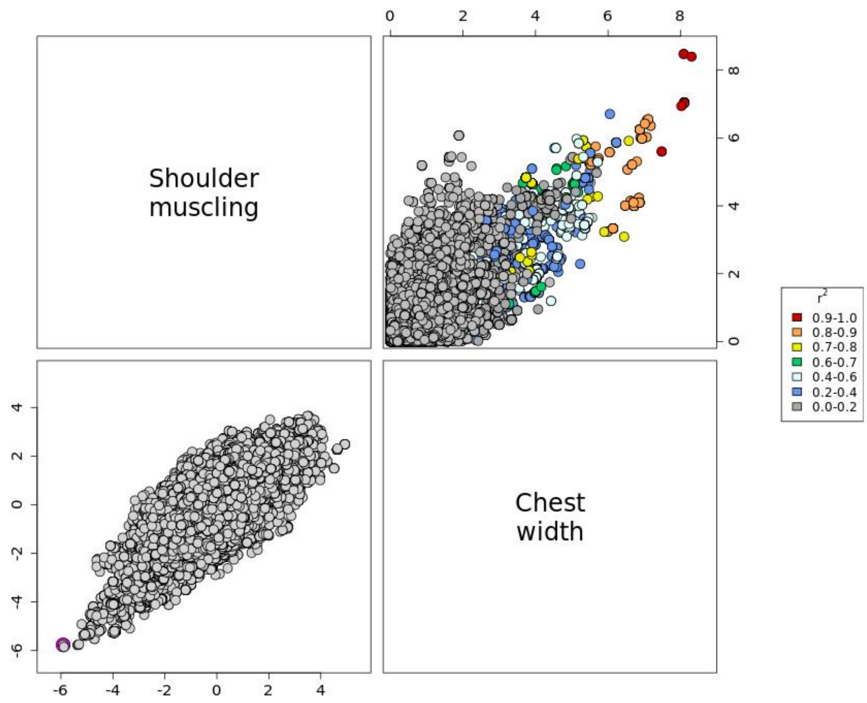

Figure S5. Scatterplots for association levels for different traits for the QTL region on BTA16. The selected traits are those harboring a significant signal in the QTLR. Upper diagonal: scatterplots with p-values on a negative log10 scale. The color represents the LD level with the lead SNP (from the trait with the strongest association). Lower diagonal: scatterplots with signed t-values. The magenta circle denotes the lead variant.

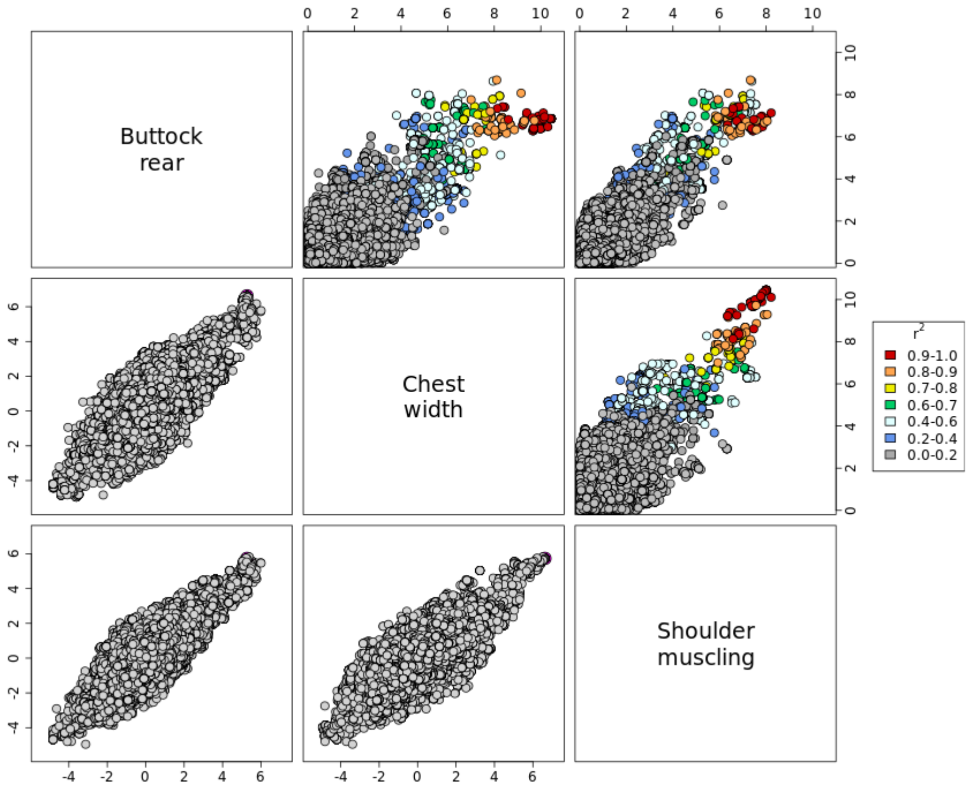

Figure S6. Scatterplots for association levels for different traits for the QTL region on BTA18. The selected traits are those harboring a significant signal in the QTLR. Upper diagonal: scatterplots with p-values on a negative log10 scale. The color represents the LD level with the lead SNP (from the trait with the strongest association). Lower diagonal: scatterplots with signed t-values. The magenta circle denotes the lead variant.

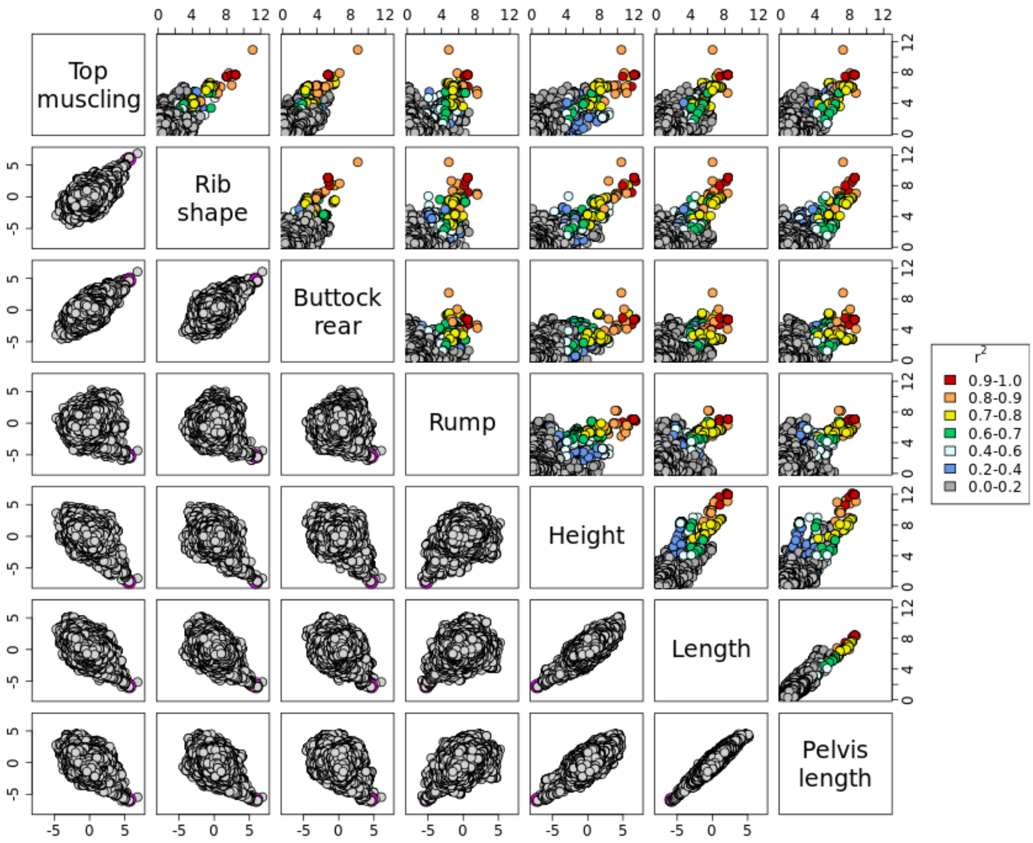

Figure S7. Scatterplots for association levels for different traits for the QTL region on BTA19. The selected traits are those harboring a significant signal in the QTLR. Upper diagonal: scatterplots with p-values on a negative log10 scale. The color represents the LD level with the lead SNP (from the trait with the strongest association). Lower diagonal: scatterplots with signed t-values. The magenta circle denotes the lead variant.

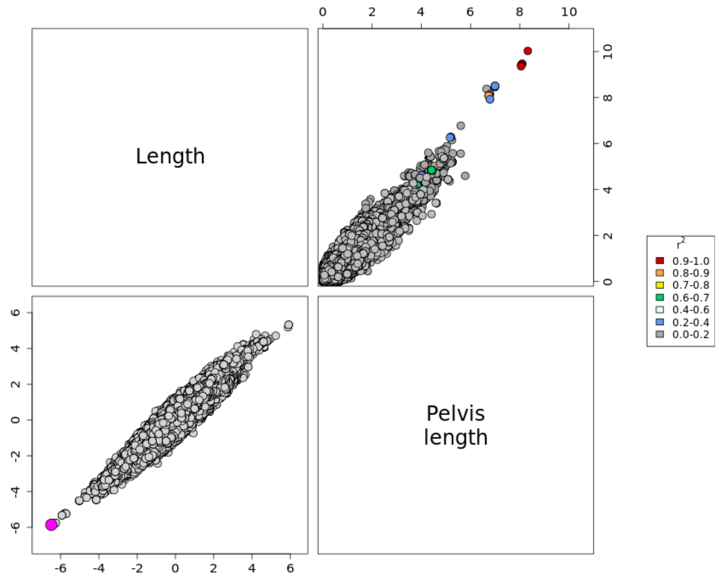

Figure S8. Scatterplots for association levels for different traits for the QTL region on BTA23. The selected traits are those harboring a significant signal in the QTLR. Upper diagonal: scatterplots with p-values on a negative log10 scale. The color represents the LD level with the lead SNP (from the trait with the strongest association). Lower diagonal: scatterplots with signed t-values. The magenta circle denotes the lead variant.

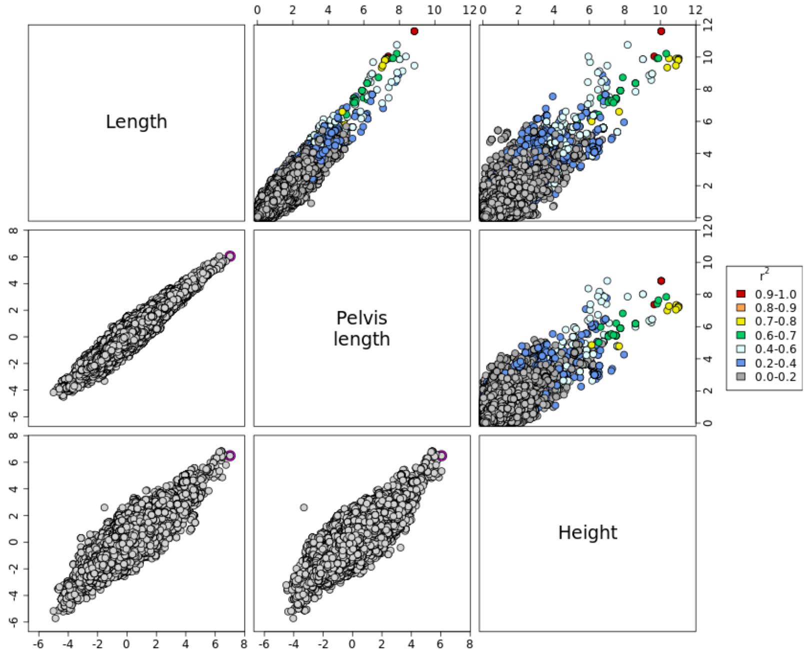

Figure S9. Scatterplots for association levels for different traits for the QTL region on BTA26. The selected traits are those harboring a significant signal in the QTLR. Upper diagonal: scatterplots with p-values on a negative log10 scale. The color represents the LD level with the lead SNP (from the trait with the strongest association). Lower diagonal: scatterplots with signed t-values. The magenta circle denotes the lead variant.

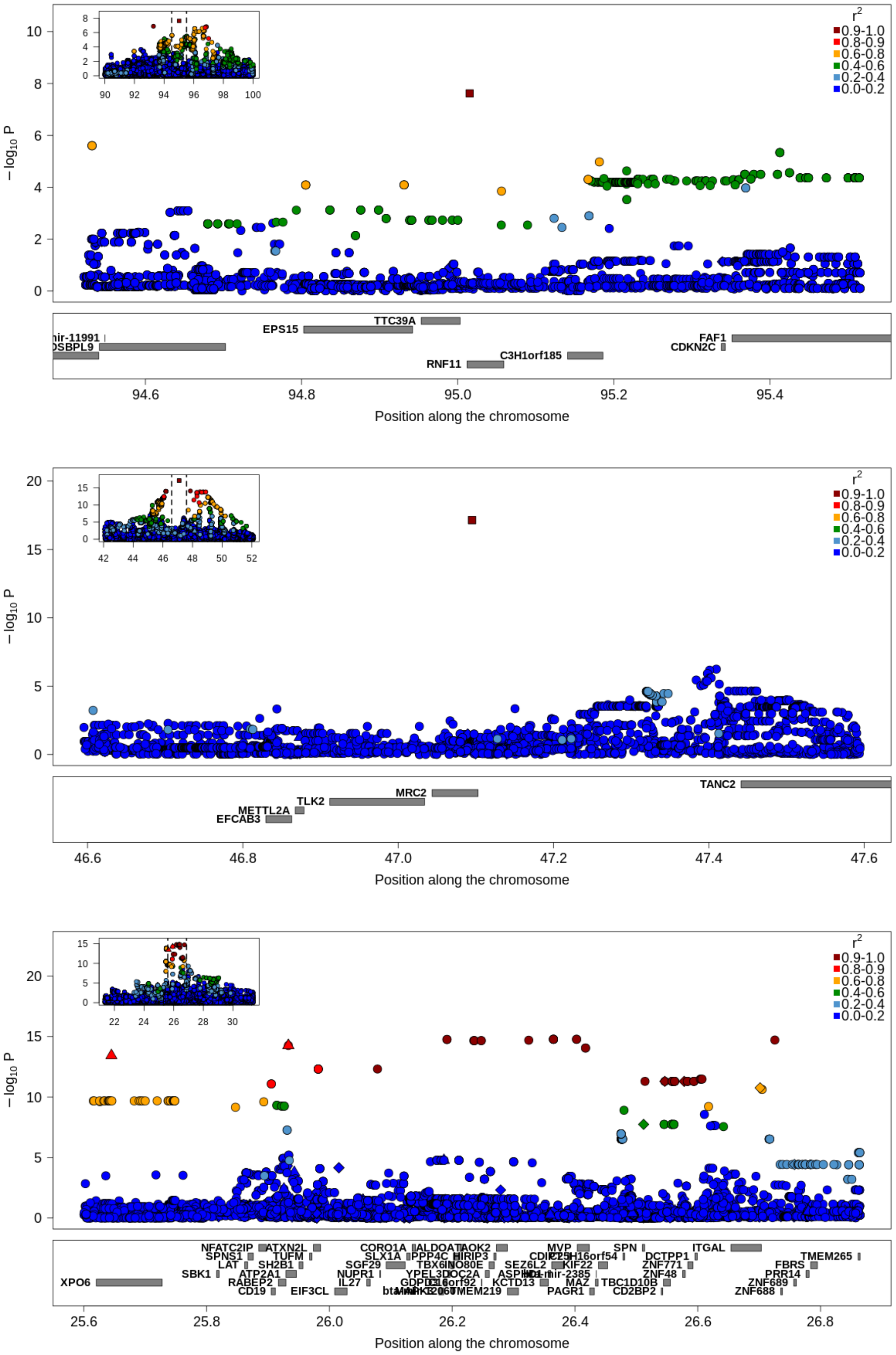

Figure S10. Regional association plot for the QTLR associated to recessive genetic defects. The colors represent the LD level with the lead variant and the symbols indicate the predicted impact of the variant (● modifier, ♦ low impact, ▲moderate impact, ■ high impact). The positions of the genes are in the lower track. Upper panel: mapping on BTA3 (MT-GWAS on muscular development traits); middle panel: mapping on BTA19 (MT-GWAS on body size traits); lower panel: mapping on BTA25 (MT-GWAS on muscular development traits).

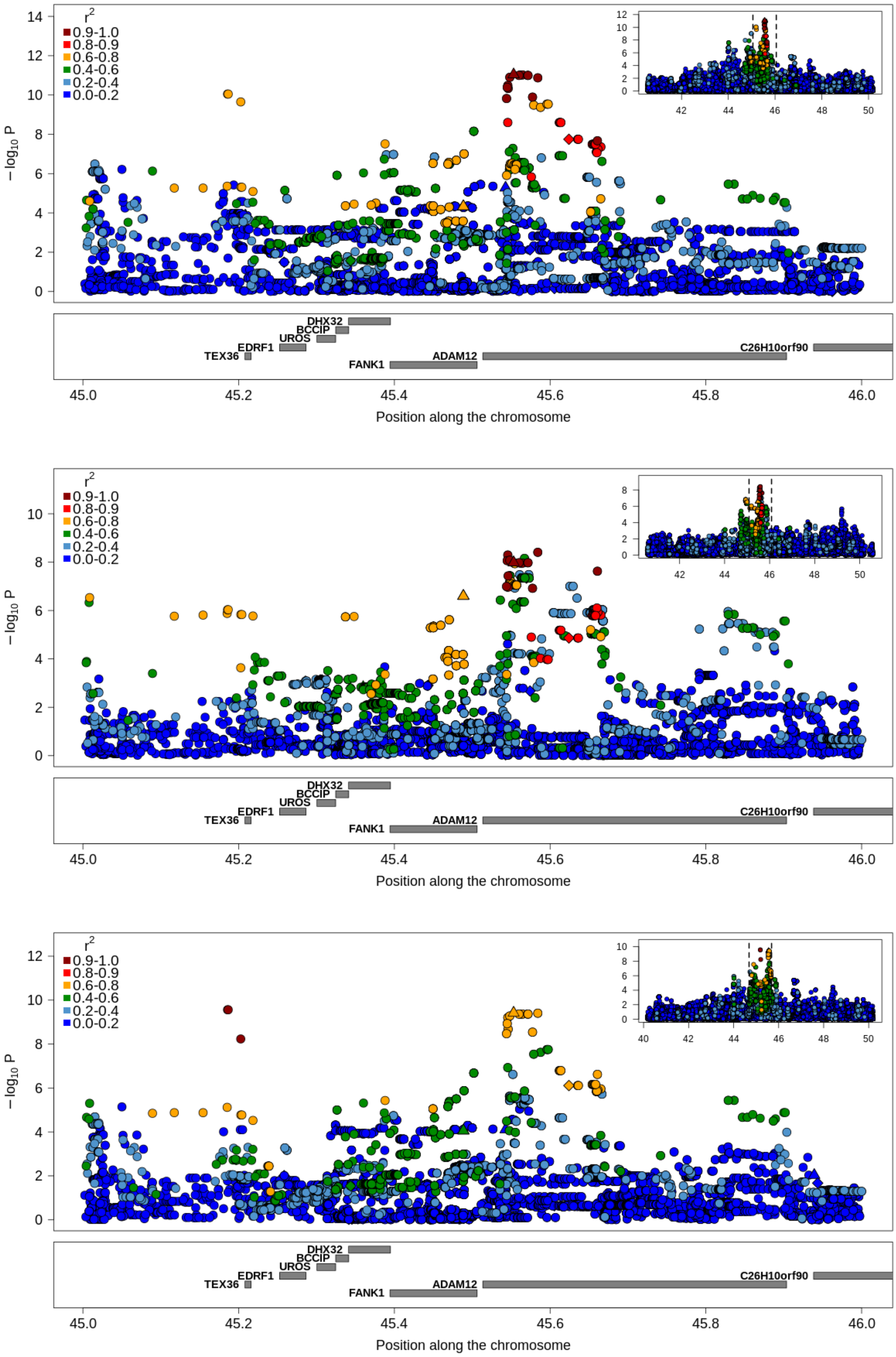

Figure S11. Regional association plot for the QTLR on BTA26. The colors represent the LD level with the lead variant and the symbols indicate the predicted impact of the variant (● modifier, ♦ low impact, ▲moderate impact, ■ high impact). The positions of the genes are in the lower track. Upper panel: ST-GWAS for height; middle panel: MT-GWAS on muscular development traits; lower panel: MT-GWAS on body size.

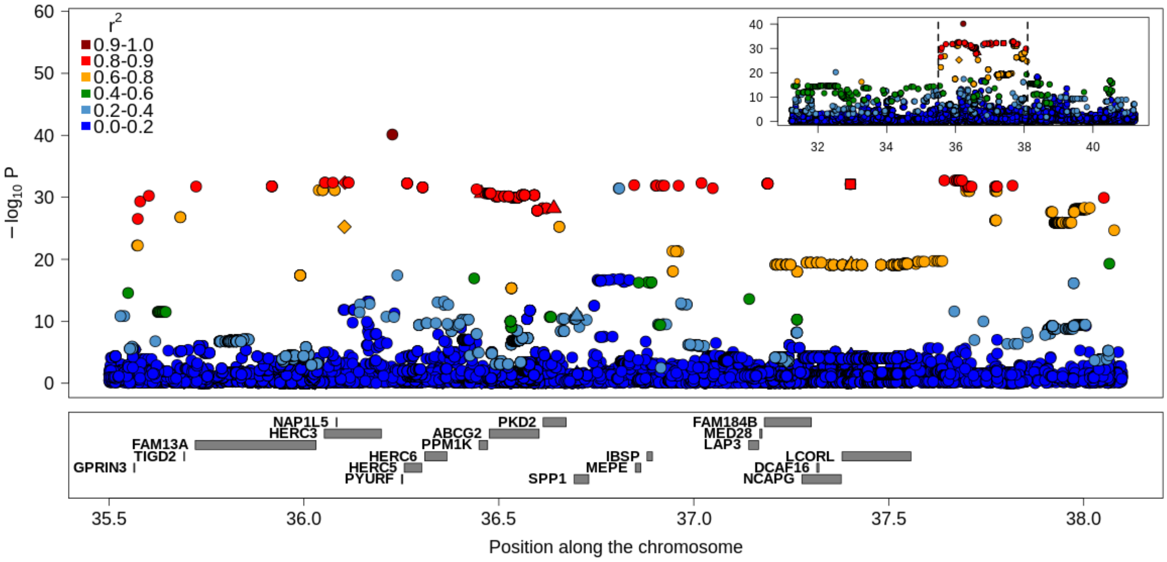

Figure S12. Regional association plot for the QTLR on BTA6. The results correspond to the MT-GWAS with traits related to body size. The colors represent the LD level with the lead variant and the symbols indicate the predicted impact of the variant (● modifier, ♦ low impact, ▲moderate impact, ■ high impact). The positions of the genes are in the lower track.

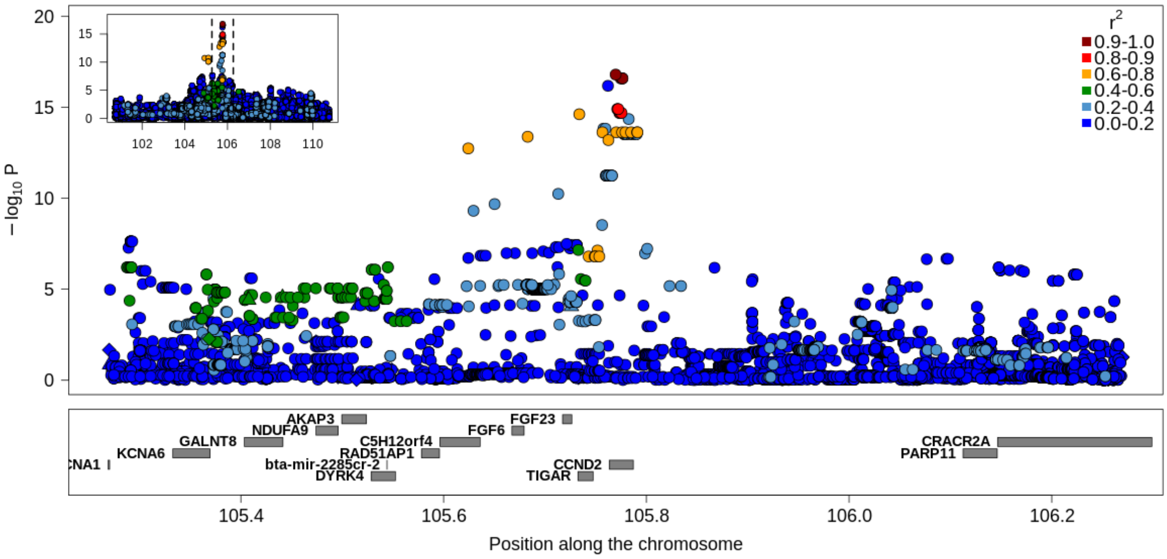

Figure S13. Regional association plot for the QTLR on BTA5. The results correspond to the MT-GWAS with traits related to body size. The colors represent the LD level with the lead variant and the symbols indicate the predicted impact of the variant (● modifier, ♦ low impact, ▲moderate impact, ■ high impact). The positions of the genes are in the lower track.

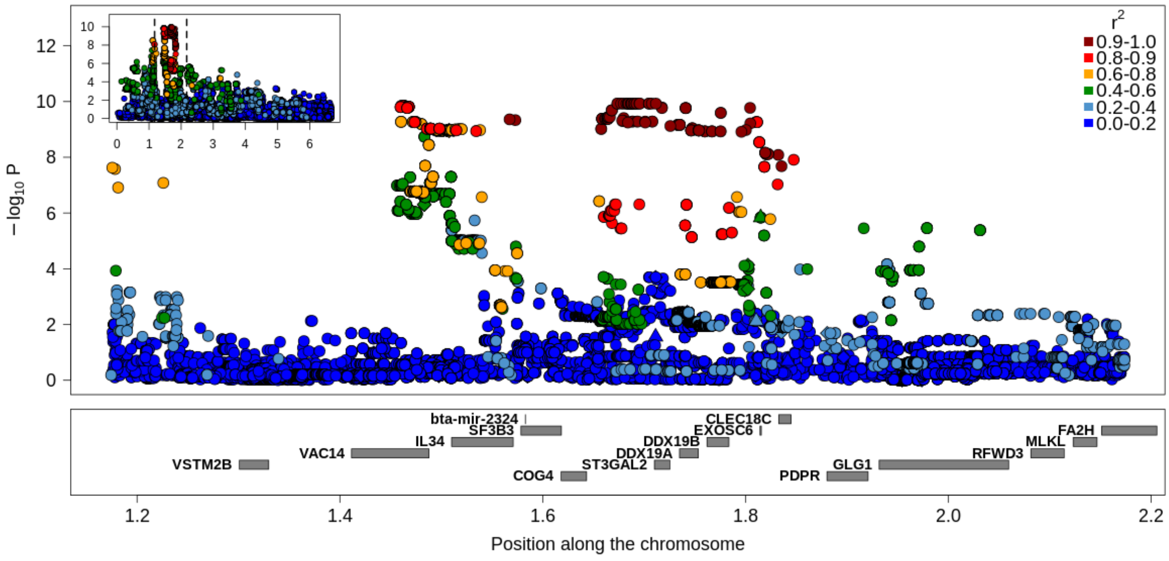

Figure S14. Regional association plot for the QTLR on BTA18. The results correspond to the MT-GWAS with traits related to body size. The colors represent the LD level with the lead variant and the symbols indicate the predicted impact of the variant (● modifier, ♦ low impact, ▲moderate impact, ■ high impact). The positions of the genes are in the lower track.

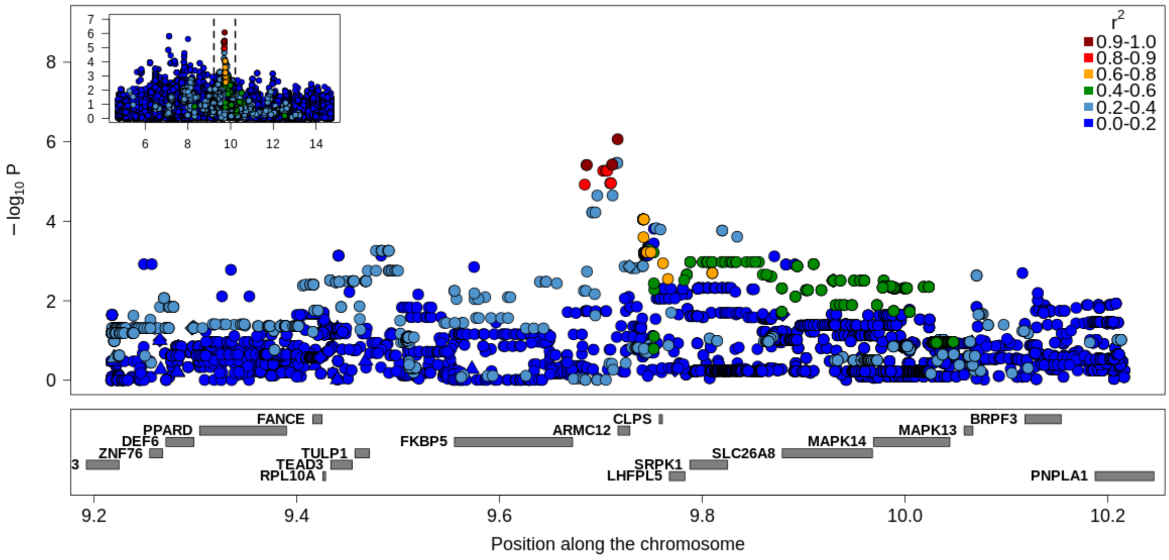

Figure S15. Regional association plot for the QTLR on BTA23. The results correspond to the MT-GWAS with traits related to body size. The colors represent the LD level with the lead variant and the symbols indicate the predicted impact of the variant (● modifier, ♦ low impact, ▲moderate impact, ■ high impact). The positions of the genes are in the lower track.

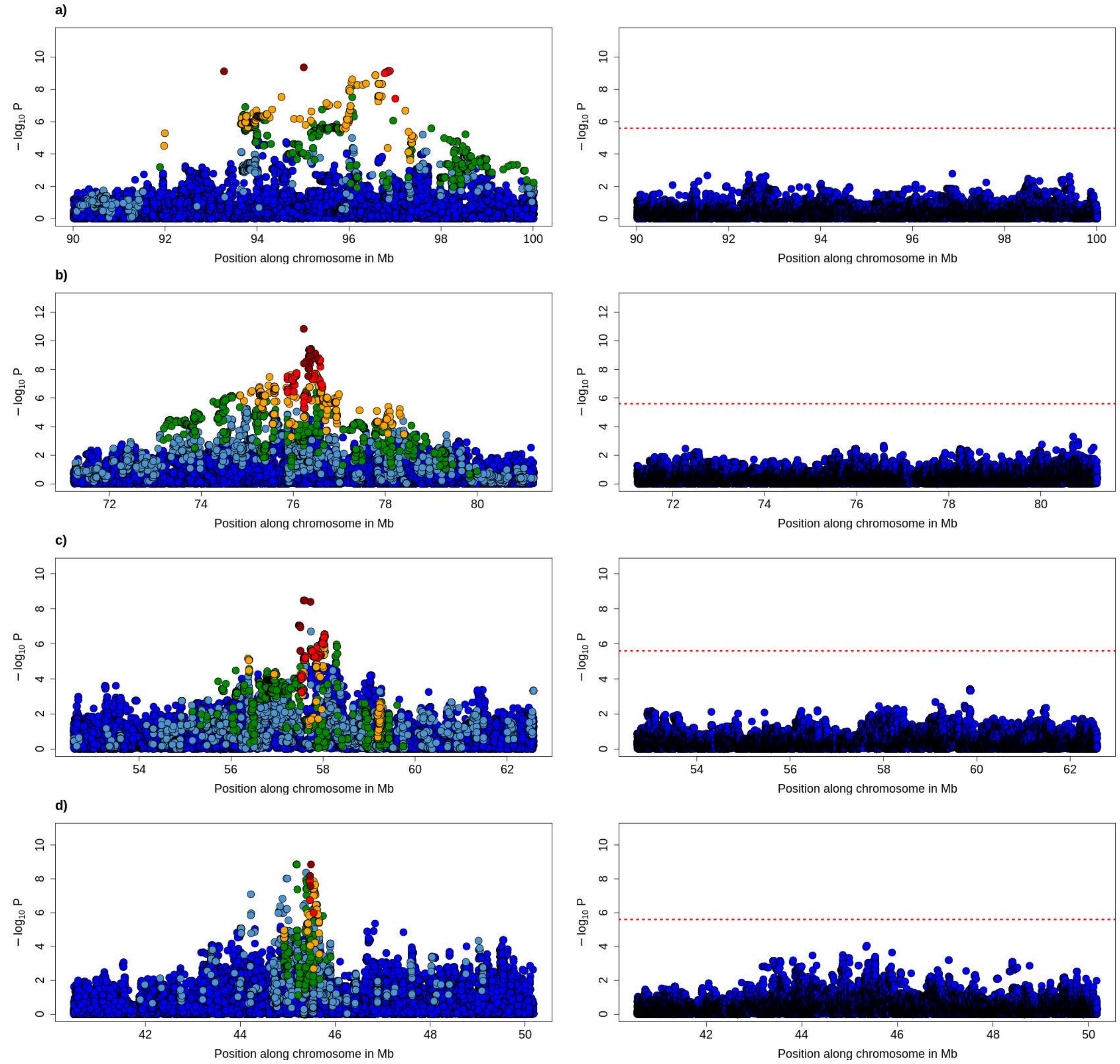

Figure S16. Regional association plot for the conditional mapping in QTLR on BTA3, BTA14, BTA16 and BTA26. The left panels represent the initial GWAS whereas the right panels correspond to conditional GWAS in which the candidate variants are fitted as covariate. The colors represent the LD level with the lead variant. The positions of the genes are in the lower track. **a**) GWAS for top muscling on BTA3, **b**) GWAS for pelvis width on BTA14, **c**) GWAS for shoulder muscling on BTA16, and **d**) GWAS for pelvis length on BTA26.

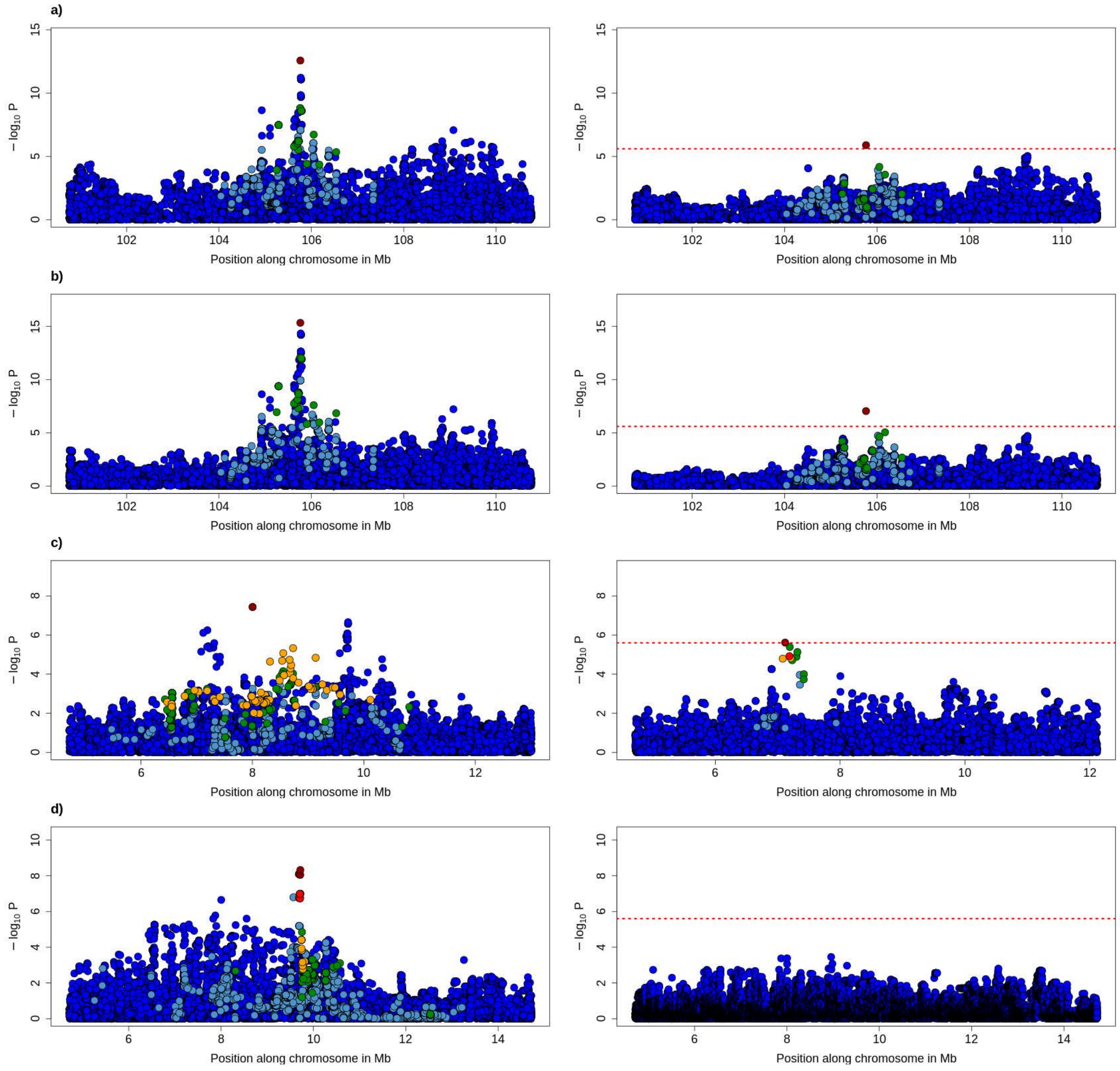

Figure S17. Regional association plot for the conditional mapping in QTLR on BTA5 and BTA23. The left panels represent the initial GWAS whereas the right panels correspond to conditional GWAS in which the candidate variants are fitted as covariate. The colors represent the LD level with the lead variant. The positions of the genes are in the lower track. **a**) GWAS for pelvis length on BTA5, **b**) GWAS for length on BTA5, **c**) GWAS for height on BTA23, and **d**) GWAS for pelvis length on BTA23.

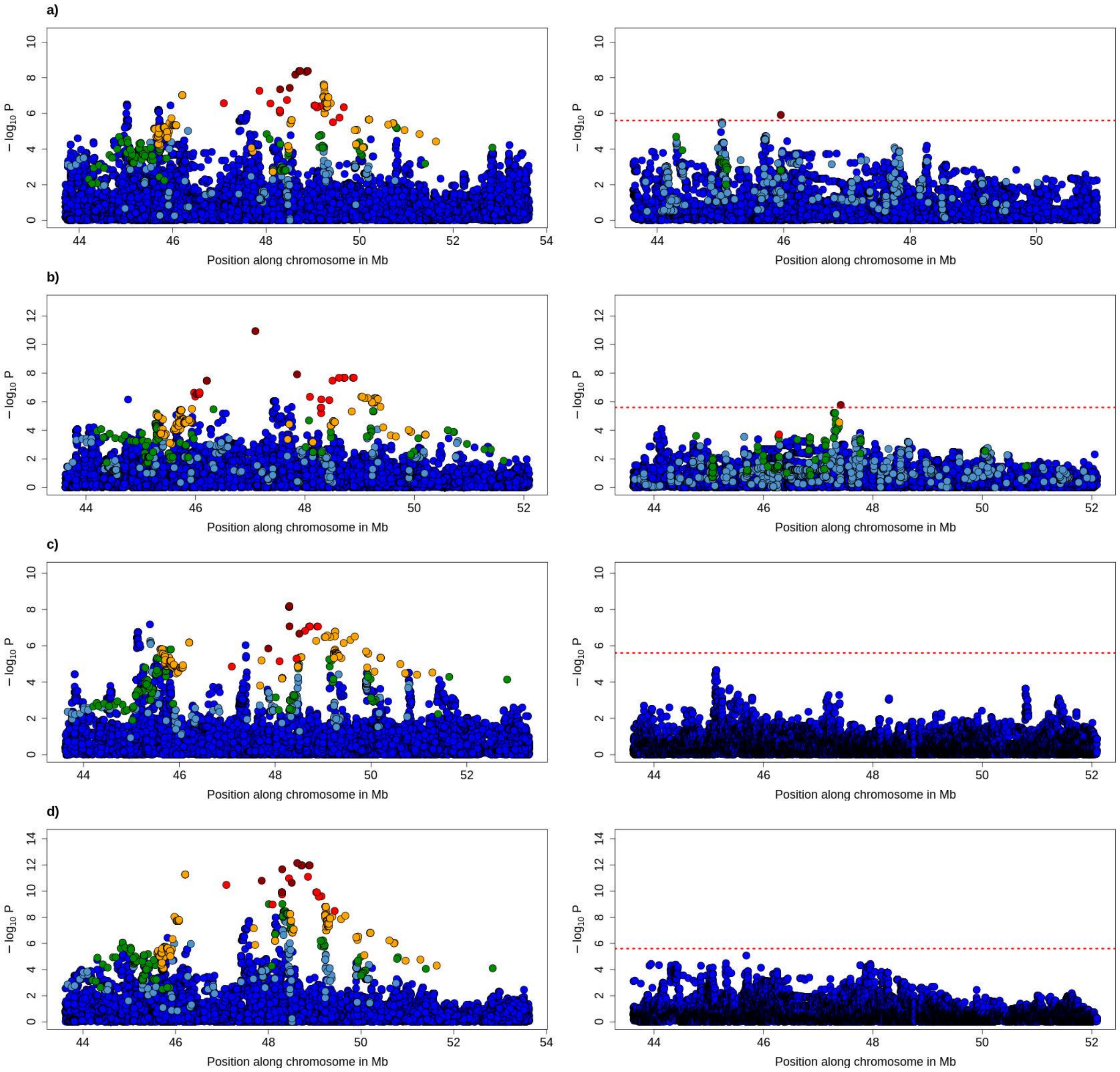

Figure S18. Regional association plot for the conditional mapping in QTLR on BTA19. The left panels represent the initial GWAS whereas the right panels correspond to conditional GWAS in which the candidate variants are fitted as covariate. The colors represent the LD level with the lead variant. The positions of the genes are in the lower track. GWAS for **a**) length, **b**) top muscling, **c**) rump, and **d**) height.

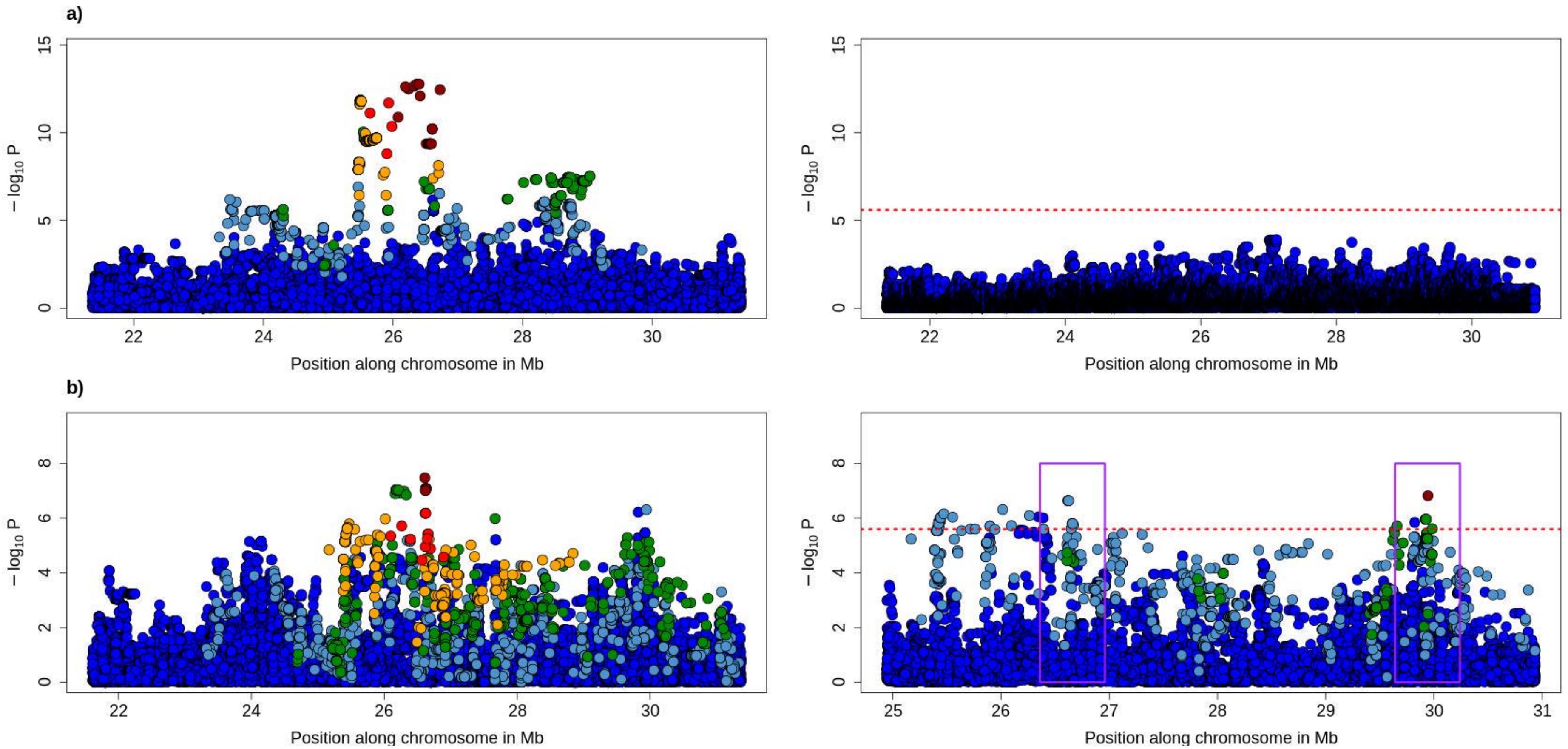

Figure S19. Regional association plot for the conditional mapping in QTLR on BTA25. The left panels represent the initial GWAS whereas the right panels correspond to conditional GWAS in which the candidate variants are fitted as covariate. The colors represent the LD level with the lead variant. The positions of the genes are in the lower track. GWAS for **a**) rump, and **b**) buttock muscling (side view). The boxes indicate the two regions achieving similar association levels and included in the credible set.

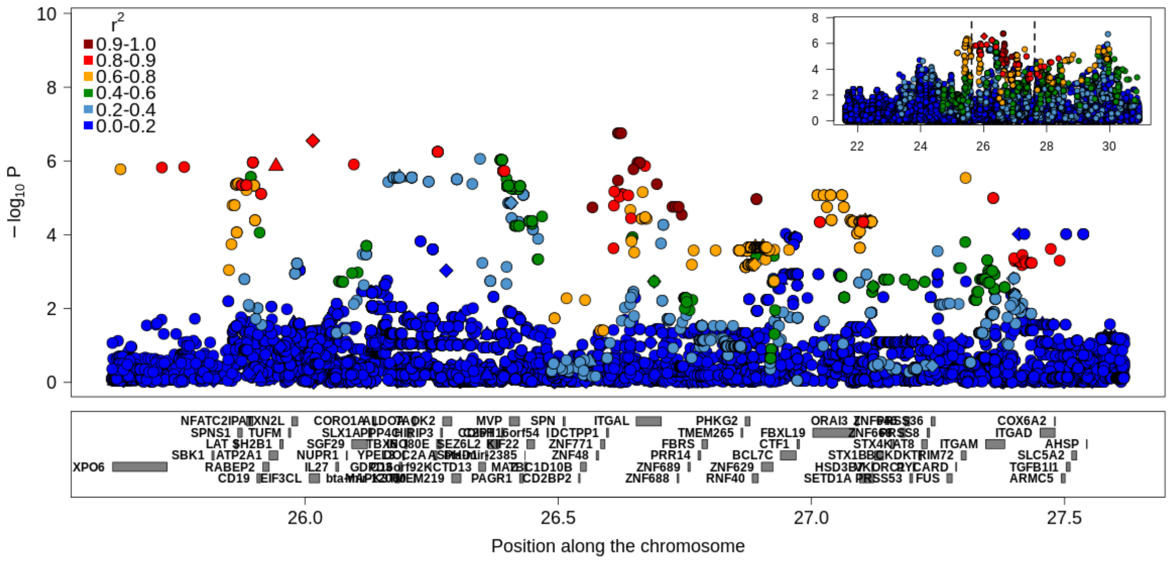

Figure S20. Regional association plot for the conditional mapping for the QTLR on BTA25. The results correspond to the MT-GWAS with traits related to buttock muscling (side view). The colors represent the LD level with the lead variant and the symbols indicate the predicted impact of the variant (● modifier, ♦ low impact, ▲moderate impact, ■ high impact). The positions of the genes are in the lower track.
