# Supplementary material for: Sequenced-based GWAS for linear classification traits in Belgian Blue beef cattle reveals new coding variants in genes regulating body size in mammals
Source: Genet Sel Evol. 2023 Nov 28;55:83. doi: 10.1186/s12711-023-00857-4 (PMC10683324; doi:10.1186/s12711-023-00857-4)
Supplement: Supplementary file 2 — Additional file 2: Table S1. Summary statistics for linear classifications traits in Belgian Blue Beef cattle. Table S2. Number of individuals genotyped on the different SNP genotyping arrays. Table S3. Description of regions included in the conditional mapping analyses. Table S4. Description of the 37 identified QTL. Table S5. Information on credible sets: number of traits sharing at least one variant in their credible set and number of traits with identical credible sets. Table S6. Comparison of LD-based credible sets (CS) and CS obtained with the IBSS approach implemented in SuSiE. Table S7. Candidate or lead variants for the 11 QTLR. Table S8. Information used for the enrichment analysis. Table S9. Most significant associations levels achieved for each trait in the conditional mapping. Table S10. Most significant associations levels achieved for traits in the second iteration of conditional mapping. [file 12711_2023_857_MOESM2_ESM.pdf]

Table S1. Summary statistics for linear classifications traits in Belgian Blue Beef cattle.

Traits	Number records	Minimum	Median	Mean	Maximum	Variance	Stand. Dev.
Length	14,476	20	39	39.3	49	4.8	2.19
Chest width	14,476	8	28	27	42	12.2	3.5
Pelvis width	14,476	11	38	38.4	45	2.6	1.61
Shoulder muscling	14,476	15	33	33.2	42	4.8	2.19
Top muscling	14,476	2	28	27.8	42	17.1	4.13
Rib shape	14,476	10	27	25.9	38	8.1	2.85
Rump	14,476	10	25	24.7	41	5.9	2.43
Pelvis length	14,476	20	39	38.7	48	4.1	2.03
Buttock side	14,476	12	38	38.4	47	3.3	1.81
Buttock rear	14,476	3	38	38.2	47	2.9	1.71
Height*	12,904	51	80	80.4	100	76.8	8.76

*Measured height is transformed in a linear score from 51 to 100. For that, the difference with the reference height measured at the same age is multiplied by 2.5. This value is then increased by 75.

Table S2. Number of individuals genotyped on the different SNP genotyping arrays.

Group of individuals	Number of individuals	Genotype array					Included in reference population	
		Illumina BovineLD	Illumina BovineSNP50	EuroG MD	Illumina BovineHD	WGS	MMD reference	HMD reference
Mapping population	11,521	x						
Mapping population	268	x	x				x	
Mapping population	2,973			x			x	
AI bulls	66	x	x				x	
AI bulls	603	x			x		x	x
AI bulls	55	x			x	x	x	x
AI bulls	57				x			x
AI bulls	2				x	x		x
Individuals without phenotype	133	x	x				x	
Individuals without phenotype	9,502			x			x	
Sequenced bulls (not in HD)	173					x		x
Total	25,353					230	13,600	890

Table S3. Description of regions included in the conditional mapping analyses

QTL-Region	Chromosome	Position on chromosome (in Mb)	Number of SNPs	Number of independent SNPs	Number of traits	Number of independent traits
1	3	90.0 – 100.0	35,494	288.1	2	1.9
2	4	106.9 – 117.9	65,663	609.1	3	2.6
3	5	101.8 – 110.8	40,782	438.8	4	2.9
4	6	31.2 – 41.2	49,140	419.3	5	3.7
5	14	71.2 – 81.2	57,569	668.8	8	4.8
6	16	52.6 – 62.6	40,014	336.2	2	1.7
7	18	0.0 – 6.7	37,802	485.5	6	4.1
8	19	43.6 – 53.6	34,361	454.5	7	5.2
9	23	4.7 – 14.7	49,894	674.5	3	2.0
10	25	21.4 – 31.4	52,314	872.2	2	1.8
11	26	40.2 – 50.2	53,432	711.6	4	2.9

Table S4. Description of the 37 identified QTL. The frequency corresponds to the allele frequency of the ALT allele.

QTL-Region*	Trait	Position (bp)	REF	ALT	Freq	p-value
BTA3:95Mb	Top muscling	95015373	T	C	0.05	4.35e-10
BTA4:112Mb	Pelvis width	111918080	T	A	0.47	5.69e-12
	Height	112030024	T	C	0.47	4.10e-10
BTA5:106Mb	Length	105762145	C	G	0.25	4.64e-16
	Pelvis length	105762145	C	G	0.25	2.69e-13
	Height	105762145	C	G	0.25	3.19e-13
BTA6:36Mb	Pelvis width	35683048	G	A	0.04	7.97e-10
	Length	36226849	A	T	0.05	1.55e-26
	Pelvis length	36226849	A	T	0.05	2.93e-16
	Height	36226849	A	T	0.05	2.41e-30
	Top muscling	37924938	G	A	0.05	3.28e-09
BTA14:76Mb	Pelvis length	75998203	G	C	0.16	7.05e-09
	Length	76227910	C	T	0.14	2.14e-10
	Chest width	76227910	C	T	0.14	5.13e-10
	Pelvis width	76227910	C	T	0.14	1.47e-11
	Shoulder muscling	76227910	C	T	0.14	4.63e-17
	Top muscling	76227910	C	T	0.14	6.30e-20
	Buttock rear	76227910	C	T	0.14	1.81e-19
	Height	76227910	C	T	0.14	6.85e-16
BTA16:58Mb	Shoulder muscling	57578954	G	A	0.72	3.35e-09
	Chest width	57725284	C	A	0.72	4.97e-09
BTA18:2Mb	Buttock rear	1165960	C	CT	0.26	2.03e-09
	Chest width	1673649	A	AT	0.27	3.59e-11
	Shoulder muscling	1717546	G	A	0.27	6.09e-09
BTA19:49Mb	Top muscling	47095175	CAG	C	0.03	1.15e-11
	Rib shape	47095175	CAG	C	0.03	8.85e-12
	Buttock rear	47095175	CAG	C	0.03	1.68e-09
	Rump	48297068	T	C	0.04	6.51e-09
	Height	48623853	G	A	0.03	7.21e-13
	Length	48707720	C	T	0.03	4.19e-09
	Pelvis length	48855548	C	G	0.04	1.70e-09
BTA23:10Mb	Length	9716619	G	A	0.48	9.30e-11
	Pelvis length	9716619	G	A	0.48	4.74e-09
BTA25:26Mb	Rump	26364983	C	T	0.01	1.68e-13
BTA26:45Mb	Length	45185751	C	T	0.62	2.47e-12
	Pelvis length	45489443	T	C	0.65	1.42e-09
	Height	45553105	G	A	0.59	9.13e-12

*The span of the QTL-Region can be found in Additional_File2.xlsx that provides the credible sets obtained for each QTL

Table S5. Information on credible sets: number of traits sharing at least one variant in their credible set and number of traits with identical credible sets.

QTL-Region	Number of traits	Number of traits with SNPs in common in the Credible Sets			Number of traits with identical SNPs in the Credible Sets		
		LD > 0.80	LD > 0.90	IBSS	LD > 0.80	LD > 0.90	IBSS
BTA3:95Mb	1	1/1	1/1	1/1	1/1	1/1	1/1
BTA4:112Mb	2	2/2	2/2	2/2	2/2	2/2	0/2
BTA5:106Mb	3	3/3	3/3	3/3	3/3	3/3	3/3
BTA6:36Mb	5	5/5	3/5	5/5	0/5	0/5	0/5
BTA14:76Mb	8	8/8	7/8	8/8	0/8	0/8	0/8
BTA16:58Mb	2	2/2	2/2	2/2	0/2	2/2	0/2
BTA18:2Mb	3	3/3	2/3	3/3	0/3	0/3	0/3
BTA19:49Mb	7	7/7	5/7	7/7	0/7	0/7	7/7
BTA23:10Mb	2	2/2	2/2	2/2	2/2	2/2	2/2
BTA25:26Mb	1	1/1	1/1	1/1	1/1	1/1	1/1
BTA26:45Mb	3	2/3	0/3	2/3	0/3	0/3	0/3

Table S6. Comparison of LD-based credible sets (CS) and CS obtained with the IBSS approach implemented in SuSiE. The threshold was set to 0.80 for the LD-based CS.

QTL-Region	Trait	Number of SNP in LD-based CS	Number of SNP in IBSS-CS	Number of SNP shared between CS
BTA3:95Mb	Top muscling	9	1	1
BTA4:112Mb	Height	72	30	30
	Pelvis width	72	30	30
BTA5:106Mb	Height	1	1	1
	Length	1	1	1
	Pelvis length	1	1	1
BTA6:36Mb	Height	74	1	1
	Length	74	1	1
	Pelvis length	74	1	1
	Pelvis width	74	30	28
	Top muscling	213	35	26
BTA14:76Mb	Height	276	101	101
	Length	276	121	121
	Pelvis length	174	174	132
	Chest width	276	132	131
	Pelvis width	276	67	67
	Buttock rear	276	34	34
	Shoulder muscling	276	81	81
	Top muscling	276	1	1
BTA16:58Mb	Chest width	105	13	13
	Shoulder muscling	120	2	2
BTA18:2Mb	Chest width	145	64	64
	Buttock rear	107	216	8
	Shoulder muscling	134	71	71
BTA19:49Mb	Height	22	1	1
	Length	24	1	1
	Pelvis length	10	1	0
	Buttock rear	29	1	1
	Top muscling	29	1	1
	Rump	16	1	1
	Rib shape	29	1	1
BTA23:10Mb	Length	13	6	6
	Pelvis length	13	6	6
BTA25:26Mb	Rump	24	8	8
BTA26:45Mb	Height	36	8	8
	Length	4	11	0
	Pelvis length	6	11	0

Table S7. Candidate or lead variants for the 11 QTLR. The table provides the number of times the variant was included in the CS (IBSS and LD-based), the number of times it was the lead variant in ST-GWAS, and the LD between the candidate variant and the lead variant in the two MT-GWAS. MT-GWAS1: MT-GWAS with traits related to height and body dimensions; MT-GWAS2: MT-GWAS with traits related to muscular development.

BTA	Position	Gene	Consequences	Presence in CS			Lead SNP in ST-GWAS	LD with lead SNP	
				IBSS	LD $r^2 > 0.90$	LD $r^2 > 0.80$		MT-GWAS1	MT-GWAS2
3	95015373	*RNF11*	Splice site variant	1/1	1/1	1/1	1/1	1	1
4	112030024	*EZH2*	Missense variant I549M	2/2	2/2	2/2	1/2	1	-
5	105769735	*CCND2*	Regulatory (ATAC-Seq, eQTL)	0/3	0/3	0/3	0/3	1	-
6	36226849		Intergenic variant	5/5	3/5	3/5	3/5	1	1
14	76227910	*WWP1*	Missense variant R844Q	7/8	7/8	7/8	7/8	1	1
16	57725284	*PAPPA2*	Missense variant P282T	1/2	2/2	2/2	1/2	1	1
18	1673649		Regulatory (ATAC-Seq)	2/3	3/3	3/3	1/3	1	1
19	47095175	*MRC2*	Frameshift variant	7/7	3/7	6/7	3/7	1	1
23	9716619	*ARMC12*	Regulatory (ATAC-Seq, eQTL)	2/2	2/2	2/2	2/2	1	-
25	25933247	*ATP2A1*	Missense variant R559C	0/1	1/1	1/1	1/1	-	0.878
26	45553105	*ADAM12*	Missense variant A582V	1/3	1/3	1/3	1/3	0.711	0.998

Table S8. Information used for the enrichment analysis. For each credible set (CS), we provide the number of variants and the number of coding variants. We performed random sampling of CS with identical number of variants and counted the number of simulations with equal or larger 1) number of coding variants, 2) number of CS with at least one coding variants, 3) number of lead SNP being a coding variant (out of 100,000,000 simulations). These counts are indicated in parenthesis below the observed values.

Credible sets (CS)	LD > 0.90		LD > 0.80	
	Num. Variants	Num. Coding Variants	Num. Variants	Num. Coding Variants
CS QTLR1 BTA3	2	1	9	1
CS QTLR2 BTA4	57	1	72	1
CS QTLR3 BTA5	6	0	14	0
CS QTLR4 BTA6	1	0	74	3
CS QTLR5 BTA14	116	1	276	1
CS QTLR6 BTA16	11	1	105	1
CS QTLR7 BTA18	86	0	145	0
CS QTLR8 BTA19	5	1	29	1
CS QTLR9 BTA23	6	0	13	0
CS QTLR10 BTA25	20	0	24	2
CS QTLR11 BTA26	18	1	43	1
Total number of variants	328	6	804	11
(Number of simulations with ≥ counts)		(10298)		(1305)
CS with at least one coding variant		6		8
(Number of simulations with ≥ counts)		(197)		(310)
CS with coding variant as lead SNP		5		5
(Number of simulations with ≥ counts)		(0)		(0)

Table S9. Most significant associations levels achieved for each trait in the conditional mapping. Association was performed only for traits presenting evidence for association in the first scan ($p < 1e-7$). Significance levels are expressed on a –log10 scale.

QTL-Region	Length	Pelvis length	Pelvis width	Chest width	Shoulder muscling	Top muscling	Buttock side	Buttock rear	Rump	Rib shape	Height
BTA3:95Mb	4.32					2.78					
BTA4:112Mb	3.19		3.09								3.46
BTA5:106Mb	7.04	5.88	5.37								5.65
BTA6:36Mb	8.08	5.27	3.92			3.61					8.77
BTA14:76Mb	3.95	4.97	3.31	4.58	3.35	3.54		3.60			5.02
BTA16:58Mb				3.35	3.41						
BTA18:2Mb				3.73	3.38	4.45	3.25	4.00			3.08
BTA19:49Mb	5.91	4.31				5.78		4.70	5.46	3.72	5.07
BTA23:10Mb	3.92	3.46									5.62
BTA25:26Mb							6.82		3.90		

Table S10. Most significant associations levels achieved for traits in the second iteration of conditional mapping. Association was performed only for traits presenting evidence for association in the conditional mapping ($p < 2.0e-6$). The significance threshold was set at $–log_{10}P > 4.7$. Significance levels are expressed on a –log10 scale.

QTL-Region	Length	Pelvis length	Buttock side	Height
BTA5:106Mb	3.39	3.76		3.57
BTA6:36Mb	4.03			2.77
BTA23:10Mb				3.80
BTA25:26Mb			3.56	
